# Supplementary material for: Factors hindering integration of care for non-communicable diseases within HIV care services in Dar es Salaam, Tanzania: The perspectives of health workers and people living with HIV
Source: PLoS One. 2021 Aug 12;16(8):e0254436. doi: 10.1371/journal.pone.0254436 (PMC8360604; doi:10.1371/journal.pone.0254436)
Supplement: S1 File — (DOCX) [file pone.0254436.s001.docx]

**SEMI-STRUCTURED INTERVIEW GUIDE FOR PLHA**

**GREETINGS**

**Do you have any of the following NCDs?**

**Hypertension, Diabetes, Cancer, Asthma, Others: a) Yes_______ b) No___________ (If the participant have none of the NCD do not proceed with the interview)**

**Where do you attend your NCD clinic?**

**a) Here at CTC_______________**

**b) Outside, other hospital or CTC____________________**

**If she/he choses (a) go to SECTIONA) and if (b) jump to SECTION B**

1. Tell me about yourself (capture socio- demographic data):

Age______________

Gender___________

Education level_____

Occupation________

Marital status______

1. **If the client receives treatment for NCD at the CTC ask the following questions**
2. Tell me more about your disease (mention e.g. cancer, diabetes, arthritis etc.)
   1. Probe more to understand when, where, how was the client diagnosed, if the diagnosis was made here at the CTC, when did she/he start NCD clinic
3. Do you get your drugs all the time you need here at CTC? ____________________
4. What are the things that makes it easy for you to get your drugs to treat______________ (mention the NCD) or to get treatment at this CTC? (Explain) (Use the probes of Why? How? Who? When where to get details. What can be done to improve that?
5. What are the things that makes it hard for you to get your drugs to treat______________ (mention the NCD) or to get treatment at this CTC? (Explain) (Use the probes of Why? How? Who? When where to get details.
6. What can be done to improve that?
7. What is your opinion if you can get all services at this clinic? (ARVs and NCD services)
8. Are you satisfied with the care for you (mention NCD that the client has) which you get from this CTC?
9. Yes / No If No please explain why (use the probes Why, How, Who) When where to get details
10. What do you advise to be done so that you will receive a better care for your NCD at this CTC?
11. Do you have anything else do you want to tell me about the services of NCD here at CTC?

**SEHEMU B: If the client does not get treatment for NCD at the CTC ask the following questions**

Tell me about yourself (capture socio- demographic data)

Age______________

Gender___________

Education level_____

Occupation________

Marital status______

1. Tell me more about your disease (mention e.g. cancer, diabetes, arthritis etc.)
   1. Probe more to understand when, where, how was the client diagnosed, if the diagnosis was made here at the CTC, when did she/he start NCD clinic
2. You said above that you don’t get treatment for_____________(mention the NCD) at this CTC. Tell me more why [use the probes of Why? How? Who? When where to get details]
3. Where do you receive treatment from? And why
4. What are the things that makes it easy for you to get your drugs to treat______________(mention the NCD) or to get treatment outside this CTC?
5. What are the things that makes it hard for you to get your drugs to treat______________(mention the NCD) or to get treatment outside this CTC? (Explain) [Use the probes of Why? How? Who? When where to get details
6. Are you satisfied with the care for your _________________ (mention the NCD that the client has) which you get from the clinic/place you are receiving treatment for now?
7. Yes / No, If no please explain why [use the probes of Why? How? Who? When where to get details
8. What would you prefer? To receive treatment for NCD at this CTC or to continue at the clinic outside this CTC where you are attending now? Tell me why
9. Do you have anything else do you want to tell me about the services of NCD here at CTC?
