# Supplementary material for: Factors hindering integration of care for non-communicable diseases within HIV care services in Dar es Salaam, Tanzania: The perspectives of health workers and people living with HIV
Source: PLoS One. 2021 Aug 12;16(8):e0254436. doi: 10.1371/journal.pone.0254436 (PMC8360604; doi:10.1371/journal.pone.0254436)
Supplement: S2 File — (DOCX) [file pone.0254436.s002.docx]

**SEMI- STRUCTURED INTERVIEW GUIDE FOR THE HEALTH WORKERS**

**Introduction:**

Interviewer: RA introduces her self

Interviewer: Tell me about yourself and your experiences (capture socio- demographic data)

1. Age
2. Level of Education
3. Work experience/duration
4. Work position
5. **Tell me do you manage NCDs (hypertension, Diabetes (DM), at this CTC?**
   1. If yes which ones (Hypertension, DM, Cancers, other chronic illnesses like asthma, renal diseases, liver etc)?
   2. If no Tell me why; do you refer patients for NCD care or do you manage them at this CTC, where do you refer? Do you get referral feedbacks?
   3. Do you think integration of NCD in HIV care is possible?
   4. IF yes explain why
   5. If no explain why
6. **What makes it easy to manage NCDs among PLWHA in this CTC?**
   1. Explain [Please use the probes of Why? How? Who? When where to get details
   2. Probe availability of drugs, lab tests?
   3. Have you been supported through trainings or mentorship on how to manage NCDs?
   4. If yes which NCDs?
   5. How many times have you received the training?
7. **What makes it hard to provide care for NCDs among PLWHA in this CTC? Please use the probes of Why? How? Who? When where to get details**
8. **Do you have existing guidelines on how to manage NCDs among PLHA?**
9. What do you recommend should be done in order to integrate care of NCDs in this CTC
10. **Is there anything we haven’t talked, about NCD you would like to add?**
