# Supplementary material for: Factors hindering integration of care for non-communicable diseases within HIV care services in Dar es Salaam, Tanzania: The perspectives of health workers and people living with HIV
Source: PLoS One. 2021 Aug 12;16(8):e0254436. doi: 10.1371/journal.pone.0254436 (PMC8360604; doi:10.1371/journal.pone.0254436)
Supplement: S3 File — (ZIP) [file pone.0254436.s003.zip › observation checklists & reports/CTC 3.docx]

**CTC 3 HOSPITAL**

**CHECKLIST NON-COMMUNICABLE DISEASES AT THE PHARMACY**

Stocks/ storage of Non communicable Diseases (NCD) drugs at pharmacy within CTC. Use this checklist to identify the availability of the following drugs

*Please put a tick ✅ if YES or NO ❌ if the drugs are unavailable*

| **S/N** | **NAME OF DRUG** | - **YES (✅)/ NO❌** |
| --- | --- | --- |
| **Antihypertensives** | | |
|  | Amlod | X |
|  | Aldoment | X |
|  | Amlodipine | X |
|  | Ascard | X |
|  | Atenolol | X |
|  | Losartam | X |
|  | Besylate | X |
|  | Clopidogrel | X |
|  | Carvedilol | X |
|  | Captopril | X |
|  | Nifedipine | X |
|  | Telmisartan | X |
|  | RepaceH | X |
|  | Lasix | X |
|  | Methyldopa | X |
|  |  |  |
| **Drugs for Diabetes** | | |
|  | Metformin | X |
|  | Ilet | X |
|  | Galvos | X |
|  | Dionil | X |
|  | Diabenese | X |
|  | Glyformin | X |
|  |  |  |
| **Drugs for cancer** | | |
|  | Chemotherapy | X |
|  | Radiotherapy |  |
|  | Leep |  |
|  | Cryotherapy |  |
| **Other NCD Drugs** | | |
|  | Aminophylline injections | X |
|  | Aminophylline | X |
|  | Aminophylline tablets | X |
|  | ARV TLD |  |
|  | Cristapen injection | X |
|  | Digoxin | X |
|  | Fluconazole | X |
|  | Ampiclox | X |
|  | Salbutamol inhaler | X |
|  | Haloperido | X |
|  | Metronidazole | X |
|  | Phenobarbital | X |
|  | **Others** |  |
|  | Condoms |  |

Name of CTC CTC 3 HOSPITAL

Client No. 1

Without being noticed, check the following services if offered within CTC. Do this for the first 20-40 PLHA. Write the word YES if vital signs are taken and NO if not. Do this for all the aspects listed below;

1. Blood pressure measured NO
2. Random Blood Glucose measured NO
3. Weight measured YES
4. Height measured. NO
5. Did you see BMI chart, BMI calculated NO

Name of CTC CTC 3 HOSPITAL

Client No. 2

Without being noticed, check the following services if offered within CTC. Do this for the first 20-40 PLHA. Write the word YES if vital signs are taken and NO if not. Do this for all the aspects listed below;

1. Blood pressure measured NO
2. Random Blood Glucose measured NO
3. Weight measured YES
4. Height measured. NO
5. Did you see BMI chart, BMI calculated NO

Name of CTC CTC 3 HOSPITAL

Client No. 3

Without being noticed, check the following services if offered within CTC. Do this for the first 20-40 PLHA. Write the word YES if vital signs are taken and NO if not. Do this for all the aspects listed below;

1. Blood pressure measured NO
2. Random Blood Glucose measured NO
3. Weight measured YES
4. Height measured. NO
5. Did you see BMI chart, BMI calculated NO

Name of CTC CTC 3 HOSPITAL

Client No. 4

Without being noticed, check the following services if offered within CTC. Do this for the first 20-40 PLHA. Write the word YES if vital signs are taken and NO if not. Do this for all the aspects listed below;

1. Blood pressure measured NO
2. Random Blood Glucose measured NO
3. Weight measured YES
4. Height measured. NO
5. Did you see BMI chart, BMI calculated NO

Name of CTC CTC 3 HOSPITAL

Client No. 5

Without being noticed, check the following services if offered within CTC. Do this for the first 20-40 PLHA. Write the word YES if vital signs are taken and NO if not. Do this for all the aspects listed below;

1. Blood pressure measured NO
2. Random Blood Glucose measured NO
3. Weight measured YES
4. Height measured. NO
5. Did you see BMI chart, BMI calculated NO

Name of CTC CTC 3 HOSPITAL

Client No. 6

Without being noticed, check the following services if offered within CTC. Do this for the first 20-40 PLHA. Write the word YES if vital signs are taken and NO if not. Do this for all the aspects listed below;

1. Blood pressure measured NO
2. Random Blood Glucose measured NO
3. Weight measured YES
4. Height measured. NO
5. Did you see BMI chart, BMI calculated NO

Name of CTC CTC 3 HOSPITAL

Client No. 7

Without being noticed, check the following services if offered within CTC. Do this for the first 20-40 PLHA. Write the word YES if vital signs are taken and NO if not. Do this for all the aspects listed below;

1. Blood pressure measured NO
2. Random Blood Glucose measured NO
3. Weight measured YES
4. Height measured. NO
5. Did you see BMI chart, BMI calculated NO

Name of CTC CTC 3 HOSPITAL

Client No. 8

Without being noticed, check the following services if offered within CTC. Do this for the first 20-40 PLHA. Write the word YES if vital signs are taken and NO if not. Do this for all the aspects listed below;

1. Blood pressure measured NO
2. Random Blood Glucose measured NO
3. Weight measured YES
4. Height measured. NO
5. Did you see BMI chart, BMI calculated NO

Name of CTC CTC 3 HOSPITAL

Client No. 9

Without being noticed, check the following services if offered within CTC. Do this for the first 20-40 PLHA. Write the word YES if vital signs are taken and NO if not. Do this for all the aspects listed below;

1. Blood pressure measured NO
2. Random Blood Glucose measured NO
3. Weight measured YES
4. Height measured. NO
5. Did you see BMI chart, BMI calculated NO
