# Supplementary material for: Factors hindering integration of care for non-communicable diseases within HIV care services in Dar es Salaam, Tanzania: The perspectives of health workers and people living with HIV
Source: PLoS One. 2021 Aug 12;16(8):e0254436. doi: 10.1371/journal.pone.0254436 (PMC8360604; doi:10.1371/journal.pone.0254436)
Supplement: S3 File — (ZIP) [file pone.0254436.s003.zip › observation checklists & reports/Checklist Vital Signs English version.docx]

**CHECKLIST FOR THE VITAL SIGNS MEASUREMENTS WITHIN CTC**

**Name of the CTC___________________________**

**No.__________________**

Without being noticed, check the following services if offered within CTC. Do this for the first 20-40 PLHA. Write the word YES if vital signs are taken and NO if not. Do this for all the aspects listed below;

1. Blood pressure measured _______________YES/ NO
2. Random Blood Glucose measured (RBG)_________________YES/NO
3. Weight measured _______________YES/NO
4. Height measured. _______________YES/NO
5. Did you see BMI chart, BMI calculated? _______________YES/NO
