# Supplementary material for: Factors hindering integration of care for non-communicable diseases within HIV care services in Dar es Salaam, Tanzania: The perspectives of health workers and people living with HIV
Source: PLoS One. 2021 Aug 12;16(8):e0254436. doi: 10.1371/journal.pone.0254436 (PMC8360604; doi:10.1371/journal.pone.0254436)
Supplement: S3 File — (ZIP) [file pone.0254436.s003.zip › observation checklists & reports/NCD Obsevation content Report..docx]

**NCD OBSERVATION REPORT**

**September 14, 2020**

A 5 days an announced onsite check-list guided observation (1 day per site) at the 5 CTCs was conducted by MDH CQI nurses from different districts. The observation was done for the 1^st^ 20-40 patients who arrived at CTCs for routine care

Each site 26 PLHA were observed from each of the 5 CTCs making a total of 130 PLHA. The nurses observed their NCD screening services they received during the visit as well as checked on the pharmacy for the availability of NCD medications. Each site was observed by a CQI nurse from a different district nurse.

**Summary of observation findings**

**Screening of PLHA for NCDs (Table 1)**

- Based on our observation in the above 5 CTCs, weight is the most commonly measured parameter for all the PLHA during routine CTC visits. It is done for all patients regardless of their types of visit (new patients or follow up patient), however none of CTCs calculated BMI for PLHA observed
- None of the CTCs did random blood sugar tests during our observation
- Only 1 out of the 5 hospitals (CTC 2) does routine measurement of blood pressure but only when the patient is suspected to have high blood pressure by the clinician
- Heights are taken for patients who are seen for the first time only and none was used to calculate BMI during our observation

**Availability of medicine NCD medications at CTCs (Table 1)**

- None of the 5 CTCs had any stoke of any form of high blood pressure (hypertension) medication (0/ 5)
- None of the 5 CTCs had in stoke any form of diabetic medication (0/5).
- Only 1 CTC (CTC 2) has medicine for cancer (chemotherapy).
- 2 CTCs had stock for other NCD medication- CTC 1 - Aminophylline tablets, and CTC 4 - Omeprazole
- Other medicine-; CTC 2, Cardisprin 75, CTC 1- Osteomin tablets

Table 1 Summary findings from onsite checklist -based observation findings of integrated HIV and NCD car practices and resources at HIV care and treatment clinics in Dar es Salaam, Tanzania in 2020 (n=130 PLHA)

| **NCD services/Drugs** | **District level CTCs** | | | | | **Total** |
| --- | --- | --- | --- | --- | --- | --- |
|  | **CTC1** | **CTC2** | **CTC3** | **CTC4** | **CTC5** |  |
| *NCD screening tests done in routine CTC visit (N=26 patients per site)* | | | | | |  |
| Blood pressure measured | 0 | 13 | 0 | 0 | 0 | 13/130 |
| Random Blood sugar done | 0 | 3 | 0 | 0 | 0 | 3/130 |
| Weight measured | 31 | 24 | 9 | 40 | 26 | 130/130 |
| Height taken | 1 | 7 | 0 | 2 | 0 | 11/130 |
| BMI calculated | 0 | 0 | 0 | 0 | 0 | 0/130 |
| *Availability of/stock of NCD drugs at CTCs (N=5 CTCs)* | | | | | |  |
| Hypertension drugs | 0 | 0 | 0 | 0 | 0 | 0/5 |
| Diabetic | 0 | 0 | 0 | 0 | 0 | 0/5 |
| Cancer chemotherapy | 0 | 1 | 0 | 0 | 0 | 1/5 |
| Other NCDs | 1 | 1 | 0 | 1 | 1 | 4/5 |
