# Supplementary material for: Factors hindering integration of care for non-communicable diseases within HIV care services in Dar es Salaam, Tanzania: The perspectives of health workers and people living with HIV
Source: PLoS One. 2021 Aug 12;16(8):e0254436. doi: 10.1371/journal.pone.0254436 (PMC8360604; doi:10.1371/journal.pone.0254436)
Supplement: S3 File — (ZIP) [file pone.0254436.s003.zip › observation checklists & reports/Checklist NCD drugs English version.docx]

**CHECK LIST NON-COMMUNICABLE DISEASES AT THE PHARMACY**

Stocks/ storage of Non communicable Diseases (NCD) Drugs at pharmacy within CTC. Use this checklist to identify the availability of the following drugs

*Please put a tick ✅ if YES or NO ❌ if the drugs are unavailable*

| **S/N** | **NAME OF A DRUG** | **YES (✅)/ NO❌** |
| --- | --- | --- |
| **Antihypertensives** | | |
|  | Amlod |  |
|  | Aldoment |  |
|  | Amlodipine |  |
|  | Ascard |  |
|  | Atenolol |  |
|  | Losartan |  |
|  | Besylate |  |
|  | Clopidogrel |  |
|  | Carvedilol |  |
|  | Captopril |  |
|  | Nifedipine |  |
|  | Telmisartan |  |
|  | Repace H |  |
|  | Lasix |  |
|  | Methyldopa |  |
| **Drugs for Diabetes** | | |
|  | Metformin |  |
|  | Ilet |  |
|  | Galvos |  |
|  | Dionil |  |
|  | Diabenese |  |
|  | Gema 2 |  |
|  | Glyformin |  |
| **Drugs for cancer** | | |
|  | Chemotherapy |  |
|  | Radiotherapy |  |
|  | Leep |  |
|  | Cryotherapy |  |
| **Other NCD Drugs** | | |
|  | Aminophylline injections |  |
|  | Aminophylline tablets |  |
|  | ARV TLD |  |
|  | Cristapen injection |  |
|  | Digoxin |  |
|  | Fluconazole |  |
|  | Ampiclox |  |
|  | Salbutamol tablets |  |
|  | Salbutamol inhaler |  |
|  | Haloperido |  |
|  | Metronidazole |  |
|  | Omeprazole |  |
|  | Phenobarbital |  |
