# Supplementary material for: Factors hindering integration of care for non-communicable diseases within HIV care services in Dar es Salaam, Tanzania: The perspectives of health workers and people living with HIV
Source: PLoS One. 2021 Aug 12;16(8):e0254436. doi: 10.1371/journal.pone.0254436 (PMC8360604; doi:10.1371/journal.pone.0254436)
Supplement: S3 File — (ZIP) [file pone.0254436.s003.zip › observation checklists & reports/CTC 1.docx]

**CTC 1 HOSPITAL**

**CHECKLIST NON-COMMUNICABLE DISEASES AT THE PHARMACY**

Stocks/ storage of Non communicable Diseases (NCD) drugs at pharmacy within CTC. Use this checklist to identify the availability of the following drugs

*Please put a tick ✅ if YES or NO ❌ if the drugs are unavailable*

| **S/N** | **NAME OF DRUG** | - **YES (✅)/ NO❌** |
| --- | --- | --- |
| **Antihypertensives** | | |
|  | Amlod | X |
|  | Aldoment | X |
|  | Amlodipine | X |
|  | Ascard | X |
|  | Atenolol | X |
|  | Losartam | X |
|  | Besylate | X |
|  | Clopidogrel | X |
|  | Carvedilol | X |
|  | Captopril | X |
|  | Nifedipine | X |
|  | Telmisartan | X |
|  | RepaceH | X |
|  | Lasix | X |
|  | Methyldopa | X |
|  |  |  |
| **Drugs for Diabetes** | | |
|  | Metformin | X |
|  | Ilet | X |
|  | Galvos | X |
|  | Dionil | X |
|  | Diabenese | X |
|  | Glyformin | X |
|  |  |  |
| **Drugs for cancer** | | |
|  | Chemotherapy | X |
|  | Radiotherapy | X |
|  | Leep | X |
|  | Cryotherapy | X |
| **Other NCD Drugs** | | |
|  | Aminophylline injections |  |
|  | Aminophylline | X |
|  | Aminophylline tablets |  |
|  | ARV TLD |  |
|  | Cristapen injection | X |
|  | Digoxin | X |
|  | Fluconazole | X |
|  | Ampiclox | X |
|  | Salbutamol inhaler | X |
|  | Haloperido | X |
|  | Metronidazole | X |
|  | Phenobarbital | X |
|  | **Others** |  |
|  | Ceptrine |  |
|  | Condoms |  |
|  | IPT |  |
|  | Osfeomin tabs (Supplements) |  |
|  |  |  |

Name of a CTC CTC 1 HOSPITAL

Client No. 1

Without being noticed, check the following services if offered within CTC. Do this for the first 20-40 PLHA. Write the word YES if vital signs are taken and NO if not. Do this for all the aspects listed below;

1. Blood pressure measured NO
2. Random Blood Glucose measured NO
3. Weight measured YES
4. Height measured. NO
5. Did you see BMI chart, BMI calculated NO

Name of a CTC CTC 1 HOSPITAL

Client No. 2

Without being noticed, check the following services if offered within CTC. Do this for the first 20-40 PLHA. Write the word YES if vital signs are taken and NO if not. Do this for all the aspects listed below;

1. Blood pressure measured NO
2. Random Blood Glucose measured NO
3. Weight measured YES
4. Height measured. NO
5. Did you see BMI chart, BMI calculated NO

Name of a CTC CTC 1 HOSPITAL

Client No. 3

Without being noticed, check the following services if offered within CTC. Do this for the first 20-40 PLHA. Write the word YES if vital signs are taken and NO if not. Do this for all the aspects listed below;

1. Blood pressure measured NO
2. Random Blood Glucose measured NO
3. Weight measured YES
4. Height measured. NO
5. Did you see BMI chart, BMI calculated NO

Name of a CTC CTC 1 HOSPITAL

Client No. 4

Without being noticed, check the following services if offered within CTC. Do this for the first 20-40 PLHA. Write the word YES if vital signs are taken and NO if not. Do this for all the aspects listed below;

1. Blood pressure measured NO
2. Random Blood Glucose measured NO
3. Weight measured YES
4. Height measured. NO
5. Did you see BMI chart, BMI calculated NO

Name of a CTC CTC 1 HOSPITAL

Client No. 5

Without being noticed, check the following services if offered within CTC. Do this for the first 20-40 PLHA. Write the word YES if vital signs are taken and NO if not. Do this for all the aspects listed below;

1. Blood pressure measured NO
2. Random Blood Glucose measured NO
3. Weight measured YES
4. Height measured. NO
5. Did you see BMI chart, BMI calculated NO

Name of a CTC CTC 1 HOSPITAL

Client No. 6

Without being noticed, check the following services if offered within CTC. Do this for the first 20-40 PLHA. Write the word YES if vital signs are taken and NO if not. Do this for all the aspects listed below;

1. Blood pressure measured NO
2. Random Blood Glucose measured NO
3. Weight measured YES
4. Height measured. NO
5. Did you see BMI chart, BMI calculated NO

Name of a CTC CTC 1 HOSPITAL

Client No. 7

Without being noticed, check the following services if offered within CTC. Do this for the first 20-40 PLHA. Write the word YES if vital signs are taken and NO if not. Do this for all the aspects listed below;

1. Blood pressure measured NO
2. Random Blood Glucose measured NO
3. Weight measured YES
4. Height measured. NO
5. Did you see BMI chart, BMI calculated NO

Name of a CTC CTC 1 HOSPITAL

Client No. 8

Without being noticed, check the following services if offered within CTC. Do this for the first 20-40 PLHA. Write the word YES if vital signs are taken and NO if not. Do this for all the aspects listed below;

1. Blood pressure measured NO
2. Random Blood Glucose measured NO
3. Weight measured YES
4. Height measured. NO
5. Did you see BMI chart, BMI calculated NO

Name of a CTC CTC 1 HOSPITAL

Client No. 9

Without being noticed, check the following services if offered within CTC. Do this for the first 20-40 PLHA. Write the word YES if vital signs are taken and NO if not. Do this for all the aspects listed below;

1. Blood pressure measured NO
2. Random Blood Glucose measured NO
3. Weight measured YES
4. Height measured. NO
5. Did you see BMI chart, BMI calculated NO

Name of a CTC CTC 1 HOSPITAL

Client No. 10

Without being noticed, check the following services if offered within CTC. Do this for the first 20-40 PLHA. Write the word YES if vital signs are taken and NO if not. Do this for all the aspects listed below;

1. Blood pressure measured NO
2. Random Blood Glucose measured NO
3. Weight measured YES
4. Height measured. NO
5. Did you see BMI chart, BMI calculated NO

Name of a CTC CTC 1 HOSPITAL

Client No. 11

Without being noticed, check the following services if offered within CTC. Do this for the first 20-40 PLHA. Write the word YES if vital signs are taken and NO if not. Do this for all the aspects listed below;

1. Blood pressure measured NO
2. Random Blood Glucose measured NO
3. Weight measured YES
4. Height measured. NO
5. Did you see BMI chart, BMI calculated NO

Name of a CTC CTC 1 HOSPITAL

Client No. 12

Without being noticed, check the following services if offered within CTC. Do this for the first 20-40 PLHA. Write the word YES if vital signs are taken and NO if not. Do this for all the aspects listed below;

1. Blood pressure measured NO
2. Random Blood Glucose measured NO
3. Weight measured YES
4. Height measured. NO
5. Did you see BMI chart, BMI calculated NO

Name of a CTC CTC 1 HOSPITAL

Client No. 13

Without being noticed, check the following services if offered within CTC. Do this for the first 20-40 PLHA. Write the word YES if vital signs are taken and NO if not. Do this for all the aspects listed below;

1. Blood pressure measured NO
2. Random Blood Glucose measured NO
3. Weight measured YES
4. Height measured. NO
5. Did you see BMI chart, BMI calculated NO

Name of a CTC CTC 1 HOSPITAL

Client No. 14

Without being noticed, check the following services if offered within CTC. Do this for the first 20-40 PLHA. Write the word YES if vital signs are taken and NO if not. Do this for all the aspects listed below;

1. Blood pressure measured NO
2. Random Blood Glucose measured NO
3. Weight measured YES
4. Height measured. NO
5. Did you see BMI chart, BMI calculated NO

Name of a CTC CTC 1 HOSPITAL

Client No. 15

Without being noticed, check the following services if offered within CTC. Do this for the first 20-40 PLHA. Write the word YES if vital signs are taken and NO if not. Do this for all the aspects listed below;

1. Blood pressure measured NO
2. Random Blood Glucose measured NO
3. Weight measured YES
4. Height measured. NO
5. Did you see BMI chart, BMI calculated NO

Name of a CTC CTC 1 HOSPITAL

Client No. 16

Without being noticed, check the following services if offered within CTC. Do this for the first 20-40 PLHA. Write the word YES if vital signs are taken and NO if not. Do this for all the aspects listed below;

1. Blood pressure measured NO
2. Random Blood Glucose measured NO
3. Weight measured YES
4. Height measured. NO
5. Did you see BMI chart, BMI calculated NO

Name of a CTC CTC 1 HOSPITAL

Client No. 17 **(New client)**

Without being noticed, check the following services if offered within CTC. Do this for the first 20-40 PLHA. Write the word YES if vital signs are taken and NO if not. Do this for all the aspects listed below;

1. Blood pressure measured NO
2. Random Blood Glucose measured NO
3. Weight measured YES
4. Height measured. YES
5. Did you see BMI chart, BMI calculated NO

Name of a CTC CTC 1 HOSPITAL

Client No. 18

Without being noticed, check the following services if offered within CTC. Do this for the first 20-40 PLHA. Write the word YES if vital signs are taken and NO if not. Do this for all the aspects listed below;

1. Blood pressure measured NO
2. Random Blood Glucose measured NO
3. Weight measured YES
4. Height measured. NO
5. Did you see BMI chart, BMI calculated NO

Name of a CTC CTC 1 HOSPITAL

Client No. 19

Without being noticed, check the following services if offered within CTC. Do this for the first 20-40 PLHA. Write the word YES if vital signs are taken and NO if not. Do this for all the aspects listed below;

1. Blood pressure measured NO
2. Random Blood Glucose measured NO
3. Weight measured YES
4. Height measured. NO
5. Did you see BMI chart, BMI calculated NO

Name of a CTC CTC 1 HOSPITAL

Client No. 20

Without being noticed, check the following services if offered within CTC. Do this for the first 20-40 PLHA. Write the word YES if vital signs are taken and NO if not. Do this for all the aspects listed below;

1. Blood pressure measured NO
2. Random Blood Glucose measured NO
3. Weight measured YES
4. Height measured. NO
5. Did you see BMI chart, BMI calculated NO

Name of a CTC CTC 1 HOSPITAL

Client No. 21

Without being noticed, check the following services if offered within CTC. Do this for the first 20-40 PLHA. Write the word YES if vital signs are taken and NO if not. Do this for all the aspects listed below;

1. Blood pressure measured NO
2. Random Blood Glucose measured NO
3. Weight measured YES
4. Height measured. NO
5. Did you see BMI chart, BMI calculated NO

Name of a CTC CTC 1 HOSPITAL

Client No. 22

Without being noticed, check the following services if offered within CTC. Do this for the first 20-40 PLHA. Write the word YES if vital signs are taken and NO if not. Do this for all the aspects listed below;

1. Blood pressure measured NO
2. Random Blood Glucose measured NO
3. Weight measured YES
4. Height measured. NO
5. Did you see BMI chart, BMI calculated NO

Name of a CTC CTC 1 HOSPITAL

Client No. 23

Without being noticed, check the following services if offered within CTC. Do this for the first 20-40 PLHA. Write the word YES if vital signs are taken and NO if not. Do this for all the aspects listed below;

1. Blood pressure measured NO
2. Random Blood Glucose measured NO
3. Weight measured YES
4. Height measured. NO
5. Did you see BMI chart, BMI calculated NO

Name of a CTC CTC 1 HOSPITAL

Client No. 24

Without being noticed, check the following services if offered within CTC. Do this for the first 20-40 PLHA. Write the word YES if vital signs are taken and NO if not. Do this for all the aspects listed below;

1. Blood pressure measured NO
2. Random Blood Glucose measured NO
3. Weight measured YES
4. Height measured. NO
5. Did you see BMI chart, BMI calculated NO

Name of a CTC CTC 1 HOSPITAL

Client No. 25

Without being noticed, check the following services if offered within CTC. Do this for the first 20-40 PLHA. Write the word YES if vital signs are taken and NO if not. Do this for all the aspects listed below;

1. Blood pressure measured NO
2. Random Blood Glucose measured NO
3. Weight measured YES
4. Height measured. NO
5. Did you see BMI chart, BMI calculated NO

Name of a CTC CTC 1 HOSPITAL

Client No. 26

Without being noticed, check the following services if offered within CTC. Do this for the first 20-40 PLHA. Write the word YES if vital signs are taken and NO if not. Do this for all the aspects listed below;

1. Blood pressure measured NO
2. Random Blood Glucose measured NO
3. Weight measured YES
4. Height measured. NO
5. Did you see BMI chart, BMI calculated NO

Name of a CTC CTC 1 HOSPITAL

Client No. 27

Without being noticed, check the following services if offered within CTC. Do this for the first 20-40 PLHA. Write the word YES if vital signs are taken and NO if not. Do this for all the aspects listed below;

1. Blood pressure measured NO
2. Random Blood Glucose measured NO
3. Weight measured YES
4. Height measured. NO
5. Did you see BMI chart, BMI calculated NO

Name of a CTC CTC 1 HOSPITAL

Client No. 28

Without being noticed, check the following services if offered within CTC. Do this for the first 20-40 PLHA. Write the word YES if vital signs are taken and NO if not. Do this for all the aspects listed below;

1. Blood pressure measured NO
2. Random Blood Glucose measured NO
3. Weight measured YES
4. Height measured. NO
5. Did you see BMI chart, BMI calculated NO

Name of a CTC CTC 1 HOSPITAL

Client No. 29

Without being noticed, check the following services if offered within CTC. Do this for the first 20-40 PLHA. Write the word YES if vital signs are taken and NO if not. Do this for all the aspects listed below;

1. Blood pressure measured NO
2. Random Blood Glucose measured NO
3. Weight measured YES
4. Height measured. NO
5. Did you see BMI chart, BMI calculated NO

Name of a CTC CTC 1 HOSPITAL

Client No. 30

Without being noticed, check the following services if offered within CTC. Do this for the first 20-40 PLHA. Write the word YES if vital signs are taken and NO if not. Do this for all the aspects listed below;

1. Blood pressure measured NO
2. Random Blood Glucose measured NO
3. Weight measured YES
4. Height measured. NO
5. Did you see BMI chart, BMI calculated NO

Name of a CTC CTC 1 HOSPITAL

Client No. 31

Without being noticed, check the following services if offered within CTC. Do this for the first 20-40 PLHA. Write the word YES if vital signs are taken and NO if not. Do this for all the aspects listed below;

1. Blood pressure measured NO
2. Random Blood Glucose measured NO
3. Weight measured YES
4. Height measured. NO
5. Did you see BMI chart, BMI calculated NO
