# Supplementary material for: Factors hindering integration of care for non-communicable diseases within HIV care services in Dar es Salaam, Tanzania: The perspectives of health workers and people living with HIV
Source: PLoS One. 2021 Aug 12;16(8):e0254436. doi: 10.1371/journal.pone.0254436 (PMC8360604; doi:10.1371/journal.pone.0254436)
Supplement: S4 File — (ZIP) [file pone.0254436.s004.zip › Transcripts PLHA/CTC2 21.docx]

**IDI:** NCD STUDY FOR HIV PLWHA

**SITE:** TEMEKE REGIONAL REFFERAL HOSPITAL

**INTERVIEWER**: L. L

**EDUCATION LEVEL:** FORM ONE

**JOB TITTLE:** BUSINESS

**MARITAL STATUS:** DIVORCED

**SEX:** FEMALE

**AGE:** 63YRS

**TYPE OF DISEASE**: HYPERTENSION AND DIABETES.

**I:** Kindly tell me, what facilitates or hinders access to care of NCDs within CTC and what can be done to improve it?

Re: Ahaa.

I: For example the way you receive your ARVs treatment here at CTC, I want to you to tell me what brings challenges in access to treatment on the side of diseases like pressure and sugar?

Re: To be honest the challenge we face, like I myself every time I visit am supposed to see a specialist, at least a doctor for diabetes. But now when you reach there they take your card and fill in their forms. They ask what problem you have. And you tell them I have diabetes and I was asking to see a doctor for diabetes. Then they can ask you who your doctor was. You tell them it is Bonny, ooh sit, these days people don’t go there as the doctors are just here. But when you enter inside you find the same normal doctors who treat normal diseases. The good thing is that I walk with an exercise book for records for sugar treatments.

[I asked her to turn up the volume]

I always walk with my book for keeping records of my sugar problem treatment. You can find sometimes I have too much numbness, sometimes dizziness and sometimes the veins pull up. Sometimes the head also aches, now if you tell them they take that exercise book and the drugs then write…..you know there are times those tins do not show properly. They write you those drugs for sugar, the ones for pressure and the ones for veins pull up, there is no more that appointment that when they run out you come and see the doctor. There is nothing….But hey tell you they are giving you a one month doze and if you take them twice a day it is 60 tablets. That means you strain by yourself, if you see they are running out you come for refill again. Then it’s like you don’t get a doctor for sugar problem and know your problems in deepness.

I: Ahaa.

Re: For example you may tell them you are feeling dizzy, nausea and am vomiting. Now if the doctor is for diabetes then they can prescribe you with tests like you go and check this….kidney or that thing. And that is how we were doing with Bony. After you have told him your legs are like swelling, at times I feel numb, too much numbness in the legs. So this is a challenge. We were supposed to have a doctor especially for diabetes. Like for us who have been long-term patients where we were taken I had a file. Every when I come I give my card and they look in their shelves and get the file. They take it to the doctor and look into records, where I started until where I have reached but now there is no that routine. Right now if you reach there they ask you patient for…. I am having sugar so they will check that sugar, they will check your pressure, and they will ask you which doctor you want to see. You enter inside. When you enter you meet a doctor in the history of your diseases you have never met .You find the ladies there and they receive your card well. They ask for your information and you give them. Sometimes they treat you due to the experience for the drugs you are taking. As far as I know every after a certain time there are changes in treatment, they don’t know. For example the day before yesterday I was prescribed some medicine and I was given the ones which when I take they don’t help me and for them they had already entered it in the system. I argued with them and in the end I avoid to be seen as stubborn. I told them to give me a little bit of them and the other ones then so that I leave. Now where would I go to tell on them?

I: When you say they had entered in the system you mean they had entered money in the system?

Re: Eeeh, yes,

I: Okay,

Re: And all of that is wrong because they came to ask me what medication I am taking while time had already gone. I showed them the cover then they tell me they are available…they just have plenty…those ones are not available. I told them they would have told me as there are many pharmacies, I would have….now you are giving me drugs that will not help me what for?

I: And it is the pharmacy here at Temeke?

Re: That one there. Just outside, if you go outside its just there.

I: So what do you advice should be done for better treatment?

Re: They should truly look into this with kindness and also carefully. They should bring us a unit….for example us people of insurance, they should get us a doctor who will be checking us and giving us treatment… Eeeh when you come to see the doctor you tell them about yourself more and because they will understanding the problem for example you have a swollen leg they already know the reason why it is swollen, Is it because of missing medication or there is a medication you have taken and does not suit you. They are understanding, for these fellows of mine they just prescribe you. Whether it reacts or not they just prescribe you. Again they must have a routine of doing us check-up quite often especially or internal organs like kidneys because a diabetes doctor understands well that diabetic people do get one, two, three complications when it is high or low. They do understand and after you have told them they get a real picture.

I: So in short mama your clinic is here at Temeke?

Re: My clinic is here at Temeke.

I: Okay.

Re: And this clinic is stopped, we have been removed from here. We are treated where other normal patients are treated.

I: Mmh… Okay, can you explain where, how and when you were diagnosed with…..you have pressure and sugar right?

Re: Yes.

I: Now where, when and how were you diagnosed?

Re: For me I came to be diagnosed with sugar in 2005.

[Answering out of the study contents]

I: Okay mama, you will tell me about your story after we are done with the interview.

Re: Okay, this disease was diagnosed in 2005 after some every now and then frustrations I started feeling tired and my heart beating very fast. After I saw that I went to Kizuiani and when I told them they gave me referral to come to Temeke. After the referral to Temeke I was checked up and found out it was 12.After I knew it was 12 they started giving me medications and by then there was no even a room for treating patients but there was just a container. It’s that container we were using. So since then I began coming to the clinic. They would give me two weeks and when my drugs are out I come back. Then later on pressure started revealing, they gave me drugs for pressure and I kept using them. I could get some funeral news and if I didn’t receive the news calmly I would faint.

I: And after all that which year was it?

Re: It was from 2005 but now I thank God.

[Out of the concept].

I: Do you get all your treatments for sugar and pressure here at your clinic?

Re: Yes, they give me.

I: They give you. And do they give you every time you want them?

Re: When they run out they give me a monthly dose then I come again, register and they give me.

I: Okay, so what is your opinion about receiving all your treatments at the clinic where you are attending now? Meaning your ARV drugs, pressure and sugar drugs. How do you see if you get them all in one place?

Re: It will look good.

I: What do you mean by saying it will look good?

Re: For example to day I came here and last Friday I was down there. The date for this clinic had not yet reached, I can’t mix them…eeeh.

I: Okay, do you say dates differ?

Re: Yes they differ.

I: Doesn’t it give you challenges by coming this day for sugar and pressure and the other day for ARVs?

Re: It is challenging for example yesterday I slept without taking my drugs, I thought when I come here they wouldn’t agree to give me all my drugs as my other visit is just tomorrow. They wouldn’t agree because they want you to come on your exact day unless you are a miss-up.

I: So what are the things that make it easy for you to get your drugs to treat your pressure and sugar diseases here at this CTC?

Re: I think it would have been nice if I could get them all here.

I: But for now is it easy for maybe or is it difficult?

Re: In?

I: Getting all your drugs here at CTC?

Re: Yes there is difficulty because the dates are different. For example today is 25^th^, and there I take drugs for the whole month and if you think you should come in the middle of the month the system reads….they tell you that you were given drugs for the whole month so it’s not your date. You see.....You can’t get these drugs.

I: So in short it doesn’t make it easy for you?

Re: To do what?

I: To get your drugs in this CTC and ……

Re: I see some difficulty, especially there.

I: It is difficult there.

Re: First of all here I can get a specialist for sugar the one who can do me a nice dialogue and may even propose for me to do some tests and do me a body check-up. Because there it’s like I just go for prescription and when am done they give me a pile of drugs. There is no one to ask how you feel after taking these drugs or what….They would just check that sugar and pressure, you go to the doctor and they prescribe drugs. And you will have a routine of reminding them that I am taking these drugs and those ones.

I: And on the side of CTC, there are no challenges?

Re: On this side, I see there are no challenges. They are just going well.

[Conversation out of the study contents]

I: May be I should ask in case you have any additions, on the side of things that you think they bring challenges for you to have access to get your drugs for pressure and sugar?

Re: What I said there is that the main problem is I have not seen specialists for diabetes. There are specialists for ears and specialists for ……but for sugar I have not seen them. Doctors were there in a diabetes unit, they were putting us patients who had insurance and the normal ones but for now those with insurance and the ones that do not use insurance are mixed in one building. I have never met a diabetes doctor to ask me whenever I enter there.

I: Okay, so are you satisfied with services you receive at CTC?

Re: Yes, I am truly grateful.

I: There are no problems…

Re: When we reach there they check us up, they serve us. Except for queue which is normal.

I: Apart from queue, any other challenge?

Re: There is none.

I: Okay,

I: Okay, so what is your opinion about receiving better treatments for pressure and sugar here at CTC?

Re: Ahaa now…for treatments for sugar together with pressure….for here….is that they should bring us a doctor who will be seeing us all. After you have [Phone interruption] come from explaining yourself….or a specialist with all the problems [Phone interruption] you see…who will be able to……to listen to us diabetic patients and pressure, and advise us, and question us. You can tell about yourself….for example we used to tell them my legs are swollen [Phone interruption] they tell you no….go check the kidneys and I go check the kidneys. And that is good, doing check-up often and not that when you go for check-up you find they have reached a bad condition, the problem has become chronic. Like the way you may find you have all the big diseases you worse. But when you do check-up when the legs are swollen or numbness….they write you to go check…go check certain things [Phone interruption] you see? Once you are told to go for check-up you check the kidneys and ….. may be heart, you check a lot of things. You live well knowing that I right now have a certain problem and am supposed to live this way ….Instead you just come and be prescribed sugar drugs mmh and you keep taking them you don’t know how your internals are. May be when my legs are swelling is because of sugar and what did you do to that sugar and what are you supposed to do? So I had a request if it’s possible they bring us doctors for us with double double problems we take our drugs here instead of having troubles….you go down there and when you go down there you find there is no a doctor for sugar. The claim that there are doctors here but I have not seen any doctor here who ask in-depth questions about sugar.

I: Okay mama, I have heard you and thank you. We have reached the end of our interview.

Re: Thank you.
