# Supplementary material for: Factors hindering integration of care for non-communicable diseases within HIV care services in Dar es Salaam, Tanzania: The perspectives of health workers and people living with HIV
Source: PLoS One. 2021 Aug 12;16(8):e0254436. doi: 10.1371/journal.pone.0254436 (PMC8360604; doi:10.1371/journal.pone.0254436)
Supplement: S4 File — (ZIP) [file pone.0254436.s004.zip › Transcripts PLHA/CTC4 0I docx.docx]

NCD STUDY: HIV PATIENT

LOCATION: MWANANYAMALA

INTERVIWER: D. K

PATIENT: 01

I: Welcome …

P: Hello.

I: Can you please raise your voice a little bit so that I can here you?

P: Okay.

I: Okay. As I explained to you before we started, my name is Diana, I want to ask you a little bit about your experience concerning treatment for…you said it is Blood Pressure…?

P: Yes, Blood Pressure

I: …okay, at this CTC in Mwananyamala. Could you start by telling me your full name, you age, your education level; so that I can get a little knowledge on your background.

P: Okay. My name is (…). My age is 48 years now.

I: Okay. Are you married?

P: Yes, I am married.

I: Okay and what work do you do at the moment?

P: I am a business woman.

I: A business woman. Okay.

And do you get treatment for blood pressure here at Mwananyamala CTC?

P: No.

I: No. Okay.

P: I have never been treated here, but I usually get treated at Dar Group…

I: Dar Group Hospital?

P: Yes.

I: Which is located where?

P: At Tazara, near Mfugale Bridge.

I: Okay. Why do you not get treated here?

P: Blood pressure did not start recently, I have had blood pressure since 2003…

I: Okay. And what hospital were you diagnosed with blood pressure at?

P: Muhimbili.

I: Muhimbili?

P: Yes.

I: And then what happened?

P: I was found to have blood pressure when I pregnant in 2003…

I: Okay.

P: …that is how it started. I was eight months pregnant…

I: Okay.

P: …and unfortunately, my blood pressure when to high and I even lost the baby; because my blood pressure went very high then dropped very low…

I: Oh, sorry.

P: I lost the child but I survived, but I was very sick…

I: Okay.

P: …after that is started to attend blood pressure clinic from then on after being found out to have blood pressure. But during all my previous children I did not have a blood pressure problem, my last child is when I got blood pressure and I lost it on 25^th^ September 2003.

I: Okay.

P: Then I attended clinic at Muhimbili and used (could not hear name of medication)

I: Okay.

P: When I completed 3 months I started feeling better; but it was not completely better because I would stay two months without any blood pressure and then later on it would come back, and it has been going on since 2003 until now.

I: Okay. So that is when you started attending the Dar Group Hospital?

P: Yes, Dar Group Hospital, I started that clinic this year….

I: Okay. So here at the CTC….

P: …I told them here the day before yesterday that I had blood pressure problems and the doctor told me to test for blood pressure and diabetes, and If I can recall it was 6 and blood pressure was 162 but usually it rises until 240 – 230.

I: Okay. But normally you go to Dar Group Hospital?

P: Yes.

I: you have never attended clinic here except for the day before yesterday when you were tested?

P: Yes.

I: And what eases your ability to get medication and treatment for blood pressure outside of this CTC?

P: Please repeat the question.

I: What makes it easy for you to get blood pressure medication or blood pressure treatment outside of this CTC clinic?

P: There is nothing that makes it easier because Dar Group is a paying type of hospital…

I: Okay.

P: …and even when it comes to the medication I have to buy it with my own money…

I: Okay.

P: …there is no preference…

I: Okay.

P: And my medication is very expensive, and I go to an expensive hospital, I pay and I buy medication with cash.

I: Okay.

And are you satisfied with the services your get regarding blood pressure treatment at the clinic that you are currently attending?

P: I do not know how to respond to you because it is not that I am satisfied, but because I want to get better that is why I go, but If there were another one other than that one which would help me live better and my blood pressure to be stable; then I have no reason to go there. But I go there because I want to live! Were I to say I do not get treated it would be a problem for me.

I: Okay.

And what would you prefer, to get your treatment for blood pressure here at the CTC or to continue getting treatment at Dar Group Hospital?

P: No. The main thing for me to get treatment. So, if I were to get treatment here it is okay, because I want to be okay. I am not picky about where I get treatment, because if I were picky I do not think it will make any sense because what I want is to be healthy; so, I get treatment here, I get treatment there; but the main thing is that I get treatment here.

I: Okay. And what do you advise should be done so that you can get better blood pressure service here at Mwananyamala CTC?

P: Meaning??

I: In your opinion?

P: In my opinion, I would like for us to get services here. Because if I am being treated at Mwananyamala it becomes easier if I come to Mwananyamala for other treatments and to check my health here; and it would make it easier for me.

I: Okay. Do you have anything to add regarding the treatment of your Blood pressure?

P: In addition, perhaps I would like to say we get assistance for frequent testing, even if it were per week because blood pressure is a disease that needs frequent testing, diabetes is a disease that need frequent testing; not like other diseases; so most importantly what I wanted to advise is that we get assistance in getting tested frequently, because blood pressure is not predictable. You can wake up and feel fine but after 3-4 hours you feel bad. So, I was asking if it is possible, for them to check blood pressure and diabetes frequently.

I: Here at the CTC?

P: …TC?..

I: CTC?? Mwananyamala Clinic.

P: Yes, Mwananyamala.

I: Okay thank you those were my questions. Thank you very much for participating.

P: Okay.
