# Supplementary material for: Factors hindering integration of care for non-communicable diseases within HIV care services in Dar es Salaam, Tanzania: The perspectives of health workers and people living with HIV
Source: PLoS One. 2021 Aug 12;16(8):e0254436. doi: 10.1371/journal.pone.0254436 (PMC8360604; doi:10.1371/journal.pone.0254436)
Supplement: S4 File — (ZIP) [file pone.0254436.s004.zip › Transcripts PLHA/CTC5 09.docx]

NCD STUDY: DIABETES AND HYPERTENSION

LOCATION: MWANANYAMALA

INTERVIWER: D K

PATIENT: 09

I: Hello, my name is Diana, I am from MDH. I have come to interview you regarding non-communicable diseases here at Mwananyamala CTC and your experience here regarding your non-communicable disease.

P: Okay.

I: First could you tell me your name, your age, your education level, are you married, are you working?

P: My name is (…) (started speaking at a low voice)

I: Could you please speak a little louder?

P: [laughs] or should I remove my face mask?

I: Even that is fine…[laughing]

P: I am (…), I am 43 years old.

I: Okay. And are you married?

P: I was married but I am now a widow.

I: Okay, and what is your education level?

P: I ended at grade 7.

I: Grade 7. Okay. And what work do you do now (…)?

P: I am just an entrepreneur. I do small jobs here and there. I braid hair…

I: Okay. So, you have a non-communicable disease, what disease is it?

P: I have Diabetes.

I: Diabetes. Okay. And do you receive Diabetes treatment here at Mwananyamala CTC?

P: Yes.

I: Can you please explain to me further regarding how you receive this treatment, how you were diagnosed…

P: I was diagnosed a long time ago. I got sick then I was admitted to Mara District Hospital

I: Okay. So not here at this CTC?

P: Yes. So, when I moved [Dar es Salaam District], I came here [Mwananyamala CTC] after becoming a widow apparently my partner had been infected [HIV/AIDS] and I was not aware, so I came to get tested and then had to join [CTC]….

I: Okay.

P: …after joining, I told them I also have Diabetes, and I then started attending the clinics and they gave me medication.

I: and how long ago was that?

P: taking medication?

I: no, when you started getting treatment…

P: I have a year and a few months…it was about two years ago…around July…

I: Okay. And do you get your Diabetes medication here at this CTC?

P: yes.

I: Okay, and what is your opinion on getting all your services together here at this clinic?

P: It would be good.

I: Okay.

P: Because one could then take out one day from working and come here you get your ARV medication and also get your diabetes medication; it would be much better…

I: Okay.

P: … instead of going clinic on different days and wasting time.

I: So where do you normally attend your diabetes clinic?

P: Right here.

I: Here?

P: Yes.

I: Because in your chart it says Mara…

P: I started clinic at Mara District, that is where I was diagnosed…

I: Okay

P: yesterday they asked me [CTC nurses] and I told them the same thing.

I: Okay.

P: I started using the medication there [Mara District]

I: Okay. And what are the things that assist in the ease of getting diabetes medication and treatment here at the CTC?

P: (laughs) assist how?

I: assist as in…to make it easier…

P: (laughs) how come that is a hard question?

I: or, is there anything that brings challenges or makes it hard for you to get diabetes treatment or medication here at this CTC?

P: No.

I: There is not?

P: No.

I: Okay. Are you satisfied with the Diabetes health services you receive here at this CTC?

P: Yes, I am satisfied with it.

I: Okay. And what would you advise be done in order to get better diabetes treatment here at the CTC?

P: to be done, maybe if the date were one date; if you were coming to collect you ARV medication then the clinics [diabetes and HIV/AIDS] be on the same day; that would be much better.

I: At the moment are they on different days?

P: Yes.

I: So, what is the process when they are different days?

P: Sometimes I come here every monthly regarding Diabetes, but in terms of ARV I have been given medication for six months…

I: Okay. So, in the middle you come for Diabetes but come for ARVs every six months?

P: Yes.

I: Do you have anything else to add regarding diabetes treatment here at Mwanyamala CTC?

P: I honestly do not.

I: Okay. Thank you (…)
