# Supplementary material for: Factors hindering integration of care for non-communicable diseases within HIV care services in Dar es Salaam, Tanzania: The perspectives of health workers and people living with HIV
Source: PLoS One. 2021 Aug 12;16(8):e0254436. doi: 10.1371/journal.pone.0254436 (PMC8360604; doi:10.1371/journal.pone.0254436)
Supplement: S4 File — (ZIP) [file pone.0254436.s004.zip › Transcripts PLHA/CTC2 17 rtf.rtf]

IDI TEMEKE PATIENT II
Female 
52 years
Completed standard seven 
Small business selling fruits for generating income
Staying at Kurasini shimo la udongo
Married
Attending CTC clinic at Temeke and pressure clinic at Balakx.
August 2020.


Interviewer: Welcome to our interview today, you have said earlier that your attending HIV clinic here at Temeke and pressure clinic at Balakx

Interviewer: When were you diagnosed that you have pressure

Respondent: It's almost four years now 

 Interviewer: Where did you first start you treatment for pressure

Respondent: At Balakx 

Interviewer: How are you doing now with the pressure treatment

Respondent: It is still very high and for the time that I ignored treatment like five months the situation is worse, I did not attend clinic and I don't know even were I have put the card.

Interviewer: What are the main reasons that were not able to attend clinic

Respondent: As I told you before it because of the family problems that I had to travel to the village. I was taking care of my sister she was sick she had mental issue problem since 2005 I took care of her until last year June when she died.

There was a child that I was taking care of her she did well in her o' level studies she now entered  form six on May so there were a lot of things.

Interviewer: Since you have started the treatment how did you see your health situation

Respondent: I was doing well

Interviewer: What are the reason for to you starting the clinic at Barakx

Respondent: I was diagnosed there then I started attending the clinic there too

Interviewer: Did you tried to switch your pressure clinic to here were you attend CTC clinic

Respondent: At the CTC clinic they said if you will be diagnosed to have another disease you will need to be treated into a different place not CTC either at other Temeke department or any other hospital outside, at the CTC they only deal with the ARVs

Interviewer: What do you think are the challenges that you met with as you attend your clinic at Temeke and Barakx 
Respondent: I did not get any challenge they attend me very well
Interviewer: What do you think are the things that contribute for the better service that you receive?

Respondent: I'm very careful with my clinic appointment dates and time for taking medication that is why even when you look at my file you find there is no any problem that is why am careful with my dose and the time that am supposed to take my dose, after I wake up and brush my teeth I take my pills I do not waste my time even when I attend clinic and have an emergence they serve me quick then I left (CTC clinic).

Interviewer: What are the indications that let you know your pressure is no good 

Respondent: When I walk my leg are turn to be weak and the serious headache that I cannot even open my eyes that I know it's a sign of pressure and when I went for testing I found out its too high but most of the time when the pressure is too high I do not even know until I get tested and found out its too high 

Interviewer: Are you satisfied with the health service that you receive at Barakx

Respondent: Yes because am paying for the medication

Interviewer: What are your opinion that can lead into service improvement 

Respondent: If I could be rich I could seek for more good service because sometime they may prescribe medication for you and you have less money that cannot afford to pay for the medication therefore you cannot take that pills  you may find you can just afford the one medication that is cheap that is the one that you're going to use, if you have been prescribed three or two medications you should take all of them together therefore you may find you have got one pill only because of the income.

Interviewer: Are you paying for the medication?

Respondent: Yes, they prescribe for you then you go and purchase there is no medication that you will be given for free.

Interviewer: At the begging you have told me that is also the government hospital

Respondent: It's a government hospital but you have to pay for everything, just for standing at the window (registration) you pay two thousands.

Interviewer: What are the things that you like more at the service that  your pressure clinic 

Respondent: Its only medication that I receive

Interviewer: On the side of CTC here at Temeke what are the things that you like mostly

Respondent: Its only medication when it's my clinic appointment I wait on the queue I take weight measurement, I  see a doctor then I take medication and some of the days when they see your file is fine you do not see the doctor rather you take your medication and you go if you have another assessment that your supposed to go through you do it like blood testing, diabetic or viral load then you do that you take the pills then you g.

Interviewer: And at the CTC clinic were you receive your HIV clinic what do you like

Respondent: They attend us very well and they talk with you friendly and listen to you carefully 

Interviewer: What are the things that you do not like?

Respondent: Nothing

Interviewer: What are your recommendations that will lead to the service improvement?

Respondent: I see everything is well done their service is very good 

Interviewer: Is there anything that you would like to share with me regarding the HIV and pressure clinic

Respondent: We receive all that when you come at the clinic they test you everything including cervix cancer, diabetes there is also prevention for TB all that are available here
It's because that when we are sick we are told to attend the pressure clinic at another place but we would like if we could receive all the treatment here that could be helpful.

Interviewer: What do you think will be more helpful?

Respondent: They could have a room for us that we get tested for pressure and all the medications here that could be good because we know here we could be more serious and they will pay more attention to us because we are weak already due to our health status but when we go to other hospitals outside we cannot afford the challenges that we are facing there that is why we do not attend the clinic on time example me am supposed to go to Barakx to start again my clinic that I stopped for five months after I travelled but I cannot go there without money therefore I should still get sick at home but if I could be treated here am sure I could be getting the medication they will just call my name XXX your pressure is high today you need to see a doctor and take medication and proceed take the pills well but here they still insist me to go to Barakx sometimes I do not have money to do that therefore the problem it keeps multiplying like now that am not feeling well and when I go to Baracks after seeing the doctor he will prescribe the medications that I cannot afford I will need to go back and the pressure will keep make me feel bad, money will still be a problem but if the service will be provided here for us who attend the HIV clinic here they could help us more .

Interviewer: Another thing


Respondent: Example I came here with my legs paining and the doctor prescribed to me medications that I can use and I went to the pharmacy for the purpose of purchasing I was not able to purchase them due to the high price and go back home and I have the prescription.

I went to ask for the medication and they told me its 45,000 and I do not have that money it means the problem persist  when I wake up in the morning my legs are paining and I cannot work properly NACHECHEMEA until after three hours then at least I feel better that is because I cannot afford to buy the medication and until now I have not purchase the pills.

Interviewer: Thank you, anything to add

Respondent: If a person is found sick here we should be treated here because we are satisfied here, we receive our pills for free because we have different economic status some they have high economic status some they have low and some very low but they could arrange for the pressure patient to be given pills and when the patient arrive with another health challenge she also receive the treatment here it could also help us and we could feel very well.

When they listen to you and you do not get the medication you feel very bad you know that you're going back home without been assisted and I cannot go back to the hospital because I do not have money I will be supposed to stay at home.

Interviewer: Pole sister, thank you for the good contribution.
