# Supplementary material for: Factors hindering integration of care for non-communicable diseases within HIV care services in Dar es Salaam, Tanzania: The perspectives of health workers and people living with HIV
Source: PLoS One. 2021 Aug 12;16(8):e0254436. doi: 10.1371/journal.pone.0254436 (PMC8360604; doi:10.1371/journal.pone.0254436)
Supplement: S4 File — (ZIP) [file pone.0254436.s004.zip › Transcripts PLHA/CTC5 13.docx]

**IDI: 1 :**NCD STUDY FOR HIV PLWHA

**SITE:** AMANA

**INTERVIEWER**: L. L

**EDUCATION LEVEL:** PRIMARY SCHOOL

**JOB TITTLE:** BUSINESS

**MARITAL STATUS:** MARRIED

**SEX:** FEMALE

**AGE:** 51YRS

**TYPE OF DISEASE**: HYPERTENSION

**I:** Kindly tell me, what facilitates or hinders access to care of NCDs within CTC and what can be done to improve it?

[Quite]

**I:** Non Communicable disease is like the one you have which is hypertention (ahaa).So what do you think hinders your access to care within CTC?

**Re:** I cannot really tell what could be the problem. I don’t really understand.

**I:** Why do you say you don’t understand?

**Re**: Because you can come here drugs and they direct you to go to there for your drugs…at the unit for hypertention patients and the drugs you are given are not the ones that always help you. They’d tell you we don’t have the other type of drugs. You’ll just have to take and proceed with them.

**I:** Is it after a check-up?

**Re**: They just check my pressure on the desk and prescribe.

**I:** And what do you think should be done?

**Re:** We are just requesting this facility to have a good stock of drugs for diseases like diabetes, pressure and the rest so that when we come here we get enough drugs and accordingly.

**I:** Do you get your treatment from this CTC? I mean for your blood pressure?

**Re**: I don’t get it here, because when I came here they told me the drugs am taking are not here at Amana.

**I**: So where do you get them from?

**Re**: For me right now I am just home, I don’t go to the hospital for my drugs anymore because where I used to go to is far. And that is where I used to go for my drugs.

**I**: Where were you getting them from?

**Re**: Rabininsia Namanga.

**I:** According to what you said previously that you don’t get your hypertention treatment at this facility (Yes) and that you got it at Rabininsia Namanga, can you explain why was it not from the facility where you always go for your CTC services?

**Re**: Rabininsia was closer to my home because I was living in Bagamoyo. I was also told that they offer good services. It’s true that they good doctors and good services. They treated us very well.

**I:** When you say they treat you well what do you mean?

**Re**: The doctor would pamper you [She laughs] though it’s a private hospital there are plenty of specialists for every kind of diseases. Whether its blood pressure or diabetes, any unit you’d want to go to.

**I:** Who introduced Rabininsia to you?

**Re**: Our neighbours at Bagamoyo are the ones who mentioned there’s nearer hospital with very good services.

**I:** When were you diagnosed with hypertention and started your clinic?

**Re:** It was either in 2015 0r 16 and I was in Moshi by that time, it was back then. I have been hypertention since I was in Moshi.

**I:** Yes.

**I:** What are the things that make it easy for you to get your drugs to treat you hypertention outside this CTC?

**Re**: It’s because of their quality services, they are very fast in searching for the drugs so that the patient gets satisfactory treatment.

**I:** So that’s what makes it easy for you?

**Re**: Yes, they are very fast according to how their hospital requires. They should not last list it as many people like it.

**I:** And you also like it?

**Re**: I also like it.

**I:** What are the things that make it hard for you to get your drugs to treat you hypertention outside this CTC?

**[**Answers out of the question**]**

**I**: I wanted to hear the challenges.

**Re**: Challenges?

**I:** On the side of Rabininsia.

**Re**: Aah challenges…for me I have not seen any challenges.

**I:** What about distance?

**Re**: For distance…yes it is far because until I reach there as from home to Bomba mbili it is 500 shillings, then to Mombasa. At Mombasa you take another transport. It’s like you must use three buses roots. So you take six roots to and fro [laughs’]. You’ll take six roots that is challenging.

**I:** No any other challenge.

**Re**: Just distance and fare. All that distance from home to there and sometimes he may tell you he [meaning the husband] doesn’t have fare while he is the one who caused this.

**I:** Are you satisfied with the care for your hypertention which you get from the place where you are receiving treatment for now?

**Re:** Yes.

**I:** What do you mean by saying you are satisfied?

**Re**: It’s the quality. (Mmh quality).I mean quality since when you reach there you take your card and go to fifth floor to wait for your doctor. That’s its quality.

**I:** Just taking the card?

**Re**: Not just taking the card because as you are waiting for the doctor since sometimes the doctor stays a bit far you are told to sit and wait for them. You find by nine or four the doctor must have come. Though if you also live far you might reach there and get the last number which could be fifty or sixty while a patient with heart disease is not supposed to get the last numbers. Even the doctor is not supposed to treat so many patients because the patient’s condition may worsen and they will have to take responsibilities.

**I:** Taking that you are satisfied with Rabininsia services, what would you prefer? To receive treatment for NCD or to continue at the clinic where you are attending now?

**I:** If the services were to be brought here we would be more relieved. So that we serve distance. We ask them to bring plenty of drugs for pressure, diabetes and many other so that it will be easier and nearer.

**[**I asked questions outside the study contents**]**

**I:** Okay, so what is your opinion about receiving all your treatments at the clinic where you are attending now?

**Re:** We just ask them to try hard to get these drugs to help us citizens with these non-communicable diseases.

**I:** You don’t have anything else to add on?

**Re:** The bad thing is am not good at talking.

**I:** Just any advice?

**Re**: We just ask the doctors for better services, more drugs so that we don’t go to other facilities.

**I:** Thank you for your time, we are done with our interview.

**Re:** Thank you.
