# Supplementary material for: Factors hindering integration of care for non-communicable diseases within HIV care services in Dar es Salaam, Tanzania: The perspectives of health workers and people living with HIV
Source: PLoS One. 2021 Aug 12;16(8):e0254436. doi: 10.1371/journal.pone.0254436 (PMC8360604; doi:10.1371/journal.pone.0254436)
Supplement: S4 File — (ZIP) [file pone.0254436.s004.zip › Transcripts PLHA/CTC5 05.docx]

NCD STUDY: DIABETES

LOCATION: MWANANYAMALA

INTERVIWER: D K

PATIENT: 05

I: I would like for you to tell me in short about yourself, your full name, your age…let us start with your full name.

P: My name is (…)i, that is my husband’s name…

I: Okay.

And what is your education level?

P: Just Form 4.

I: Are you married?

P: Yes, I am married.

I: Okay, and how old are you (…)?

P: I am 49 years old.

I: Okay. And what work do you do?

P: I used to work as a store keeper in the ministry but after my health problems I had to retire and rest…

I: okay.

P: …I was at internal affairs ministry.

I: Okay, you had told me that your non-communicable disease is Diabetes…

P: Yes.

I: Okay. Do you get Diabetes treatment at Mwananyamala CTC?

P: Yes, we come to the Mwananyamala CTC…

I: Okay...

P: …the last time during the Corona incident they had told us to stay at home until Corona finishes then we go, then during the time of quarantine that is when the condition changed…

I: Okay, so you had been getting treatment for your Diabetes at Mwananyamala?

P: Yes.

I: At the CTC clinic or somewhere else within Mwananyamala Hospital?

P: It is within Mwananyamala Hospital, on the left side near reception… [not at the CTC clinic]

I: Okay.

And were you diagnosed with Diabetes at Mwananyamala or somewhere else?

P: Somewhere else.

I: Where was it?

P: It was in Arusha.

I: And do you get your Diabetes medication at the Mwananyamala CTC?

P: They write us prescriptions and then we go buy.

I: So where do you normally go buy?

P: We go buy at a shop outside at Mwananyamala. [Pharmacy outside Mwananyamala Hospital]

I: Okay. How would you feel if you were to get all the services for Diabetes and ARV at your ARV clinic? What is your opinion regarding this??

P: I would appreciate it very much…

I: And what are the things that challenge or make it easy for you to get medication that treats Diabetes?

P: Honestly, when I am prescribed the medication, that is when I go buy them anywhere, in honestly the medication is expensive so we persevere to our best ability, if we do not have [money] sometimes we just stay as we are [do not buy medication].

I: Okay.

And are you satisfied with the Diabetes health services that you receive at Mwananyamala?

P: Honestly, I am satisfied. They handle us well and treat us well. Maybe it is just us who are unable to meet the requirements, because maybe you are told to eat this way and you do not have [nutritional requirement] so you do not take or you are told to take you medication on time, and you find you do not have or they are finished so you wait 2-3 days and then buy them and take them.

I: Okay. And what would you advise be done so that you can get better treatment for Diabetes?

P: I do not have anything to say because I have no options, if the government could find a way to help us we would be happy because we die because of not having assistance to get the medication, because sometimes you are told (Jema2 name of medication) where one pill is tshs. 1000 and you have to take one pill every morning; another one is Metaphomen, you buy them; for example, I have been told to take three small ones and one big one, you go buy them, when they are finished you start struggling…

I: Okay. And another question…I just wanted to get further clarification; the Diabetes clinic that you go to, it is not at the same place as the ARV clinic?

P: No, we go that side [gives directions to the Diabetes clinic which is in a different building from the CTC building] which is then the Diabetes clinic.

I: Okay. Thank you very much (…), that is the end of my questions thank you for your time.

P: Okay.

I: Okay.
