# Supplementary material for: Factors hindering integration of care for non-communicable diseases within HIV care services in Dar es Salaam, Tanzania: The perspectives of health workers and people living with HIV
Source: PLoS One. 2021 Aug 12;16(8):e0254436. doi: 10.1371/journal.pone.0254436 (PMC8360604; doi:10.1371/journal.pone.0254436)
Supplement: S4 File — (ZIP) [file pone.0254436.s004.zip › Transcripts PLHA/CTC5 04.docx]

NCD STUDY: DIABETES

LOCATION: MWANANYAMALA

INTERVIWER: D K

PATIENT: 04

I: Hello

P: Hello.

I: My name is Diana, I am from MDH. I have come to interview about non-communicable diseases and their treatment here at this CTC.

Please start by telling me in short, you age, your name, marital status, employment and the like.

You can start with your name.

P: Okay. My name is (…).

I: Okay. And how old are you (…)?

P: I am 69.

I: and are you married?

P: I was married but my wife has passed away.

I: Okay. And what work do you do?

P: Business activities.

I: Business activates, okay.

And what non-communicable disease do you have?

P: Diabetes, Blood Pressure.

I: Diabetes and Blood Pressure. Okay.

In your opinion what do you think influences or challenges the availability of non-communicable disease treatment at this CTC clinic?

P: Repeat.

I: What in your opinion, challenges or influences the availability of treatment of Diabetes and Blood Pressure at this CTC?

P: (grunts) That is a hard question…

I: Or let me ask you, do you get treatment for Diabetes and Blood pressure at this CTC?

P: Yes. I usually get there (indicating another building) at Insurance...

I: Where?

P: There at insurance (building where people with insurance go pay)

I: So, it is not here at this CTC?

P: No.

I: It is a different department?

P: Yes.

I: What do you think makes it difficult for you to get diabetes and blood pressure treatment at the CTC?

P: They have not put…

I: They have not....

P: They have not put that. [diabetes and blood pressure treatment].

I: Okay. And what of medication for diabetes and blood pressure? Do you get them here at this CTC or…?

P: I get it at Insurance...

I: at Insurance. Okay.

And what influences or eases the ability to get diabetes medication?

P: Influences?

I: Yes

P: Eases?

I: Yes.

P: …well when I come I fill in the form at insurance and then they give me the medication depending on which medication I am using.

I: Okay. So, when you were diagnosed with Diabetes and Blood pressure…

P: Yes.

I: were you diagnosed here at this CTC or somewhere else?

P: no, it is a long time ago.

I: Okay, what was the process until they discovered that you have diabetes and blood pressure, if you can explain to me one at a time…

P: For example, Diabetes, I had taken Quinine at a time when I had malaria, the night that I finished the dose I got an epileptic shock and I bit down on my tongue and lost consciousness and urinated on myself…

********************* INTERVIEW WAS INTERUPTED ****************************

I: Okay. Let us continue…

You were telling me about when you were discovered to have Diabetes…

********************* DISTURBANCE IN THE BACKGROUND ***********************

P: …after fainting at night and losing consciousness, they woke up in the morning and knocked on my door; I had closed my door; they then rushed me to Palestina, where they believed that I was dead because of how bad my condition was; when they wanted to take me to the intended place (mortuary), a neighbor of mine felt my veins and told them ‘this man is not dead yet, blood is still flowing in his veins’ ; then the doctor and nurse came back, put me in an ambulance and brought me here to Mwananyamala….

I: Okay. At this point had you already started attending CTC clinics here at Mwananyamala?

P: No not yet….

I: You had not been detected that you were infected?...

P: I did not have it [HIV]….

I: Okay so you got it after…

P: …. later on, years later….

I: So, you started with Diabetes and then….

P: …. yes, and blood pressure. Blood pressure after my wife died, that is when blood pressure started.

I: Okay. Ad when the blood pressure started had you began attending Mwananyamala clinic?

P: No.

I: Okay.

In your opinion what would you advise be done so that you can get better treatment for diabetes and blood pressure at this CTC clinic in Mwananyamala?

P: They should just plan that instead of going there [insurance building], they bring it to us here, it would be nice.

I: What is the process at the moment? Do you come here first then go there...?

P: …. process is just if my diabetes or blood pressure medication is I go to insurance, or I go to any other hospital and show them the diabetes and blood pressure medication I use they write for me.

I: Okay.

So normally what hospitals do you go to?

P: Normally I go to Palestina or Magomeni…

I: Okay, so it is not necessarily here at Mwananyamala?

P: In the beginning I used to go to a clinic that was at Muhimbili, but then I started realizing that was too far, and because I understand the medication, and I usually write them in a book and I also do self-tests because I have a glucometer, so if it goes up or if it goes down, I then know what to use. Because of it goes down I drink a soda and it stabilizes, it means the medication is too much. So, I self-test.

I: Okay, so you said you used to go to Muhimbili and then moved here to Mwananyamala because it was closer. Did you also attend CTC clinics at Muhimbili or here??

P: Here.

I: So here at Mwananyamala they also provide treatment for Diabetes and Blood pressure but not here at this CTC?

P: Yes.

I: But still right here at Mwananyamala?

P: Yes, right here at Mwananyamala, there is a building down there at the Insurance building…

I: Okay.

Are you satisfied with the diabetes and blood pressure health services that you receive here?

P: Yes I am satisfied because it is my responsibility to use medication on time and follow what the doctor tells me…

I: So on the days when you have CTC clinic and also a diabetes clinic; what is the process? Do you start here and then go there or there and then here?

P: Because it is nearby, I just go any time, or sometimes I start there I take medication and come here.

I: I do not think I have any more questions to ask you, thank you very much.

If you have anything else to add regarding treatment of diabetes and blood pressure here at this CTC?

P: I do not see any problem because I can go there and come here but if it is possible for them to bring it here fine, if they do not bring…I will go any place because I understand my medication…

I: …and getting the medication is no problem?

P: …there is no problem in getting the medicine, for example like these ones (opens bag and takes out medication) are finishing today…

I: …okay, what are those called?

P: Metaph..(inaudible)

I: Phoman..??

P: Metaphoman for Diabetes.

I: Metaphoman, Okay.

P: And these one are for Blood pressure.

I: and what are those called?

P: [shows box]

I: Amodepine, okay.

So they finished, and you are going to get them over there [insurance building]…

P: Yes I am going to go get. Normally when I get there they just prescribe for me.

I: Okay. Thank you. Have a good day.

P: Okay.
