# Supplementary material for: Factors hindering integration of care for non-communicable diseases within HIV care services in Dar es Salaam, Tanzania: The perspectives of health workers and people living with HIV
Source: PLoS One. 2021 Aug 12;16(8):e0254436. doi: 10.1371/journal.pone.0254436 (PMC8360604; doi:10.1371/journal.pone.0254436)
Supplement: S4 File — (ZIP) [file pone.0254436.s004.zip › Transcripts PLHA/CTC5 12.docx]

**IDI:** 12.

**SITE:** AMANA RR. HOSPITAL

**INTERVIEWER**: L L

**EDUCATION LEVEL:** .STD SEVEN

**JOB TITTLE:** HOUSE WIFE

**MARITAL STATUS:** MARRIED

**SEX:** FEMALE

**AGE:** 49YRS

**TYPE OF DISEASE**: ENLARGED HEART

**I**: Do you get your treatment for heart disease from this CTC?

**Re**: Yes.

**I:** Kindly tell me more, when and where was this NCD diagnosis made? You also tell me if it was made here.

**Re**: I did all the tests and diagnosed here. When I fell sick they brought me here and took some tests and that’s when they found out I have heart enlargement and hypotension. So I started treatment and all the services here.

**I:** What facilitates or hinders access to care of NCDs (like this heart disease) among people within CTCs and what can be done to improve it?

**[**Silence**]**

**I:** Did you understand the question?

**Re**: Yes I understood. Since we are not professionals so it is hard to know. Because we just get the services and instructions according to how the doctor directs us.

**I**: So you can’t tell what you think hinders you from getting treatment for NCDs among treatment centres for people living with HIV.

**[**Silence**]**

**Re**: To say the truth I wouldn’t know what the problem is because these drugs are very expensive and very limited.

**I:** Do you think there are limitations and something should be done for you to receive better care?

**I:** Like what kind of limitations?

**Re**: One of them is their availability in all facilities so that once you visit them you manage to get treatment. It’s hard when you go to visit your relatives and unfortunately get occupied while at the same time your clinic date has come. You may find that when you go to some facilities to look for treatment you don’t get it. So we kindly ask for every facility to have specialists to make it easy for us patients with heart disease to be treated wherever we may go.

**I:** Has it ever happened that you came here and did not get treatment.

**Re**: It has never happened.

**I:** Okay. Do you get the drugs every time you need them at this CTC?

**Re**: Yes.

**I:** There is no a day you have ever missed?

**Re**: Yes.

**I:** What is your opinion if you can get all services here at CTC? How do you see it?

**Re**: It becomes very joyful to get all the services in one place. There will be no inconveniences. See, like this time we have left our homes to come here and spent money for fare. Sometimes you find on your day of visit you don’t have fare. So if you get all the services in one place becomes much better.

**I:** What are the things that make it easy for you to get your drugs to treat you heart disease at this CTC?

**Re**: Makes it easy?

**I:** Yes, makes it easy

**Re:** Just like I said, our doctors receive us well and offer us good services.

**I**: Is it that it’s just good services that make it easy for you? Nothing else?

**[**I asked a question out of the contents**]**

**I:** What are the things that make it hard for you to get drugs to treat your heart disease at this CTC?

**Re**: What hinders me?

**I:** Yes, or give you trouble in to get your drugs here at CTC, I mean for your heart disease.

**Re**: For the heart drugs, the only problem is that they are costly and you have to pay for them. So just like I said initially. There are days you don’t have money and fail to buy them because we are not given for free.

**[**I asked a question out of the contents**]**.

**Re:** Though where I come from is very far from here there is a relief that I get to receive all my treatments under one visit. Unlike wise if I was attending HIV clinic here and hypotension clinic at another hospital.

**I:** What do you advise to be done so that you will receive a better care for your hypotension at this CTC?

**Re**: What should be done will depend on how you doctors will help us and here our cry about drugs costs. You help us at least we get them for free so that we can have a better life. One my loose life because of missing these heart drugs, because if you miss them for two day you become very weak. They get finished.

**I:** What do you mean when you say they get finished?

**Re**: It means you become seriously sick because you must have ruined the whole system of medication.

**I:** Thank you, we have reached the end of our interview.

**Re**: Thank you.
