# Supplementary material for: Factors hindering integration of care for non-communicable diseases within HIV care services in Dar es Salaam, Tanzania: The perspectives of health workers and people living with HIV
Source: PLoS One. 2021 Aug 12;16(8):e0254436. doi: 10.1371/journal.pone.0254436 (PMC8360604; doi:10.1371/journal.pone.0254436)
Supplement: S4 File — (ZIP) [file pone.0254436.s004.zip › Transcripts PLHA/CTC1 20 rtf.rtf]

Female III
CTC clinic Sinza Diabetic clinic Rabinisia
Standard seven
Small business for income generation
45years 
2017 started diabetic clinic
Married
Staying at Tegeta.
Patient is using health insurance in accessing diabetic treatment.

The patient suffered from diabetic for a long time and was wrongly diagnosed at the first time as her body was badly affected remained skinned, lose weight and went for cervix cancer testing several times then later it was diagnosed as diabetic and start the treatment.

Interviewer: Can you explain to me a bit regarding the diabetic treatment that you receive

Respondent: I'm using health insurance therefore I consult specialist, I get a good treatment and I see that he is taking a good care of me. There is no challenge in getting medication any time that I need medication I get them.

Interviewer: How about CTC side

Respondent: I get the medication also although during corona I face some challenges but now things are moving on well.

Interviewer: Apart from consulting specialist at your diabetic clinic what else are you happy at your diabetic clinic

Respondent: Hospitality. The give you hopes and encouragement there is time that I went to the clinic and I have loosed hope because the know about my HIV status it also written in the computer he gives me hope, instruct me what to be done, what to be carefully with and that is not end of the life, he treat me very well

Interviewer: What do you think can be done to improve more the service that you receive 

Respondent: If I could also attending CTC clinic at the same place am attending the diabetic clinic, you may find today am here next day am there I could save time, I wish I could have one clinic

Interviewer: Apart from serving time what else do you think you will benefit

Respondent: Even bus fare, every clinic that I attend I must use bus fare, I need also to reach at the clinic fast get the service and left like today I just eat a piece of pumpkin so am feeling hungry that is the challenge

Interviewer: What are the challenge that you most face in the two clinic that you're attending

Respondent: Truly I have never face a challenge because most of them they know me very well, because they knew before since I was skinned and boneless that I cannot even sit I used to come here and cry the viral load was very high and looked like a someone who lose hope so they are used to me as they remember my journey they thank God, am doing well.

Interviewer: How did you realized that you have diabetic

Respondent: On 2013 my sister that we were born two of us was killed by the car I don't know because of depression again on 2016 my daughter that was born with HIV I have two kids was involved in a road accident, I was admitted at Muhimbili and did not realized that I have this problem then after I found in my secret party after sexual intercourse with partner I was bleeding even when I want to check myself with a finger I found out am still bleeding then I went to see a doctor and found that the vagina area was badly affected and referred to Ocean road for cancer checkup at that time I did not check for diabetic and there was a very long series of checkup and losing weight later on I was testing for diabetic and found that am diabetic at that time I was in a serious pain, very dark and relatives were trying every options at last it was found the sugar level was 35 and it was not cancer. I used diabetic medication and now am feeling better.

Interviewer: Thank you very much.
