# Supplementary material for: Factors hindering integration of care for non-communicable diseases within HIV care services in Dar es Salaam, Tanzania: The perspectives of health workers and people living with HIV
Source: PLoS One. 2021 Aug 12;16(8):e0254436. doi: 10.1371/journal.pone.0254436 (PMC8360604; doi:10.1371/journal.pone.0254436)
Supplement: S4 File — (ZIP) [file pone.0254436.s004.zip › Transcripts PLHA/CTC5 08.docx]

NCD STUDY: HIV PATIENT

LOCATION: MWANANYAMALA

INTERVIWER: D K

PATIENT: 08

I: Okay (…) before we begin, I would like to get a little information regarding you, so if you can please tell me your full name, you age, your education level, weather you are married and work that you do. Let us start with your full name…

P: My name is the same one…

I: Could you please tell me…

P: (…)

I: Okay. How old are you?

P: 50.

I: Okay, and are you married?

P: I was married I am not divorced.

I: Okay. And what work do you do?

P: A business person.

I: Okay. In the beginning [before beginning the interview] you had told me that you have Diabetes and Blood pressure…

P: Yes.

I: …and you had said that you get treated for Diabetes and Blood pressure where…?

P: Plan hospital. It is a government hospital at Buguruni.

I: Okay, and why have you decided to get treatment for Diabetes and Blood pressure at Buguruni instead of Mwananyamala where you go get your ARVs?

P: I had originally started at Amana hospital then we were transferred to Buguruni, it is just what I decided on my own.

I: Okay. So, what did you decide to go to Buguruni instead of seeking treatment at Mwananyamala?

P: It is just what I decided.

I: So, you do not have a specific reason?

P: No.

I: And what eases your ability to get Diabetes and Blood pressure medication?

P: we just buy them, there is nothing that eases it, I just buy them.

I: So, do you get medication at Buguruni or do they write a prescription for you then you go buy?

P: I get a prescription then I go buy.

I: And are you satisfied with the treatment that you get at Buguruni for Diabetes and Blood pressure?

P: yes.

I: Okay. And which would you prefer, to get treatment for Diabetes and Blood pressure at Mwananyamala where you get your ARVs medication or to continue to get treatment at Buguruni?

P: no, it is just as I have planned it is the same thing that I will continue to do…

I: so, what if treatment for Diabetes and Blood pressure is started at Mwananyamala where you go get ARVs?

P: It’s Okay.

I: And what do you advise be done so that you get better treatment for Diabetes and Blood pressure at Mwananyamala clinic?

P: I have not understood what to be done…

I: …. what would you advise?

P: …however it will be is fine.

I: Okay. Thank you, for your time.
