# Supplementary material for: Factors hindering integration of care for non-communicable diseases within HIV care services in Dar es Salaam, Tanzania: The perspectives of health workers and people living with HIV
Source: PLoS One. 2021 Aug 12;16(8):e0254436. doi: 10.1371/journal.pone.0254436 (PMC8360604; doi:10.1371/journal.pone.0254436)
Supplement: S4 File — (ZIP) [file pone.0254436.s004.zip › Transcripts PLHA/CTC3 10.docx]

NCD STUDY: HYPERTENSION

LOCATION: VIJIBWENI

INTERVIWER: D. K

PATIENT: (…)

I: Welcome. To start with I would like to ask you to tell me you full name.

P: (…)

I: Okay.

And how old are you (…)?

P: 60.

I: And what is your education level?

P: Grade 7.

I: Are you married?

P: Yes, I am married but I do not live with my husband at the moment.

I: Okay. And what work do you do (…)y?

P: I am retired. I used to be a teacher but I am now retired.

I: Okay. And what non-communicable disease had you earlier told me you have…??

P: BP is the only non-communicable disease that I have…

I: Okay, and do you receive treatment for BP at Vijibweni CTC?

P: Yes.

I: Okay. Can you please tell me a little more regarding the treatment for BP that you get at Vijibweni CTC…?

P: I tell you more…??

I: Yes, is it the same place where you get you ARVs or….

P: …the place where I get my ARVs is different from the BP [Treatment] but it is the same hospital.

I: Okay, so please tell me how do you get your BP medication…

P: Usually when my monthly BP medication finished, I go back to the hospital and they give me.

I: And where in Vijibweni [Hospital] do they treat your BP?

P: (laughs) I do not know where to say exactly, inside the hospital in the middle near reception…

I: …Okay…

P: there is a room for BP, Diabetes and Eye diseases…

I: Okay.

So, it is not where you go collect your ARVs?

P: Yes. I go collect my ARVs at the department at the end near the Mortuary.

I: Okay. And what is your opinion regarding getting all the services together at your ARV clinic?

P: It would be much better because this month this side sees me, next month that side sees me (laughs)…

I: Okay.

And what are the things that challenge or make it easy for your Blood Pressure medication?

P: What challenges to get medication?...

I: Yes.

What makes you unable or makes it easier to get your Blood Pressure medication?

P: Honestly, I am satisfied with the service we get there, when we get there they care about us we are treated and we leave…I have not been unable to get treatment honestly…

I: Okay….

P: …which makes me certain of my trip in that when I go there I get treatment and I go back home.

I: Okay.

So, you get medication at Vijibweni?

P: Yes, inside at Vijibweni.

I: Okay…

P: …except maybe the BP medication, if they do not have, they prescribe for those of us who have health insurance and we go get them at pharmacies which are registered under health insurance.

I: Okay.

And what would you advise be done so as to get better treatment in regards to Blood Pressure at you CTC clinic, as in where you go collect your ARVs?

P: Mmhm. Firstly for us BP patients we would like to request; because at time we do not understand these BP medications, they mix our medications and do not tell us what a medication treats in your heart and how it helps us; some months you may get this type and another month another type, they just prescribe them silently and you do not understand and you just take them, later on an outsider may come and tell you ‘this medication will not help to reduce you Blood pressure’ so you find we are just buying them randomly…

I: …. Okay…

P: …so they should make sure they medication they are giving us are legitimate and tell us what it is for so that we can be keen and know what there is a certain medication I am missing.

I: Okay, so they should explain more clearly to you?

P: Yes.

I: And are you satisfied with the Blood Pressure service that you are getting at VIjibweni?

*************** INTERRUPTION ************************

I: Okay, let us continue. I was asking you weather you were satisfied with the Blood Pressure health service that you are getting at Vijibweni.

P: I am honestly satisfied with it, because I have not gotten any problems and when I take their medication I have not had any problems…

I: Okay…

P: …the service that I get there is good and they care about us, and me myself; when I tell them, they bring me good medication. When my Blood pressure go up they advise me, when it goes down they also tell me. So, I am satisfied with them.

I: Okay.

And you last opinion regarding getting Blood Pressure services at the ARV clinic that you go to at the CTC?

P: My last opinion is that I ask at least, I hear that there is a specialist heart doctor who normally comes there [NCD clinic], but some of us have never seen him. I would ask; so that we have faith in our treatment; that we are able to see him in turn, so that we have assurance that we are also okay. Because some of us have never seen him, we get treated but we do not know whether they are actually heart specialist or not…

I: Okay.

P: …. Yes, so he should also be certain that he sees all of us, so that he can tell us, and give us hope that we are doing well and the medication we are getting is good or not good so that we have faith in our Doctors treatment because even the specialist has said so. Instead of just leaving us unaware and sometimes our Blood Pressure goes up and we start thinking ‘what is wrong with our Doctor?’…

I: Okay.

Thank you very much Lucy for your time.

P: Thank you to you too for checking on us.

I: Have a good day.

P: Okay.
