# Supplementary material for: Factors hindering integration of care for non-communicable diseases within HIV care services in Dar es Salaam, Tanzania: The perspectives of health workers and people living with HIV
Source: PLoS One. 2021 Aug 12;16(8):e0254436. doi: 10.1371/journal.pone.0254436 (PMC8360604; doi:10.1371/journal.pone.0254436)
Supplement: S4 File — (ZIP) [file pone.0254436.s004.zip › Transcripts PLHA/CTC4 03docx.docx]

NCD STUDY: HYPERTENSION, ULCERS

LOCATION: MWANANYAMALA

INTERVIWER: D. K

PATIENT: 03

I: Hello (…)

P: Hello.

I: Could you please speak a little louder so that you can be heard…

P: Okay.

I: As I told you, I am from MDH, I want to interview regarding treatment you get concerning non-communicable diseases here at the CTC. But firstly, I would like for you to tell me about yourself; your name, clearly, your age, your education level, work that you do…

P: Okay.

I: Let us start with your name.

P: My name is (…)

I: How old are you Fatuma?

P: I am 57 years old

I: Okay, and are you married?

P: No, I had a partner, but he passed away since 2006; I am not just with my kids who help me.

I: Okay. And what work do you do?

P: My male child who I live with works in education…

I: Okay. You do not work?

P: I do not work. He works in the ministry.

I: And before we started recording, you had informed me that you have Blood Pressure and…please repeat the diseases you earlier stated

P: Blood Pressure, ulcers and HIV.

I: And do you receive treatment for Blood Pressure and your ulcers here’s at this CTC?

P: No, I get treatment for blood Pressure and ulcers at Mikocheni…

I: Which hospital in Mikocheni?

P: Kairuki. HIV I get treatment here at Mwananyamala.

I: Okay. And you have already mentioned that you do not get treatment for Blood Pressure and Ulcers here, can you….

P: …blood pressure and ulcers and other problems...

I: can you please tell me why you get treatment at Kairuki instead of getting treatment here?

P: I went to Kairuki before getting my problems with here with HIV…

I: So, the blood pressure started?

P: I went there [Kairuki] before I started my HIV problems. I went there and they discovered I had blood pressure and ulcers because I used to have dihorrea and my child said let us just got also test for HIV and then brought me here [Mwananyamala].

I: So, at the time when you were going to Kairuki you had not yet been infected?

P: I had not yet known what problem I have [that she was infected with HIV]

I: Okay. And they did not tell you that you have HIV?

P: No, they just told me that I have blood pressure and ulcers.

I: Okay. Why do you still go to Kairuki to receive treatment for blood Pressure and ulcers, why not get it [treatment] here at the CTC?

P: They told me that, my blood pressure is a lot, and I would have to attend clinic for blood pressure every month from the beginning over there [Kairuki] before I came here; and that I am supposed to take one pill every day and not miss a single dose. So, I used to take the medication but I still did not feel well, I was still sick; I would go to the clinic [Kairuki] and get the medication but nothing; that is when my children decided that I should come here and get tested for HIV…

I: Okay…

P: … so when I got tested here, that is when they diagnosed this problem [HIV]. So, I attend this clinic, and also go there [Kairuki] at the end of the month on the required date.

I: Okay. And you get the blood pressure medication from Kairuki?

P: Yes.

I: And are you satisfied with the treatment that you get for blood pressure from the other clinic [Kairuki]?

P: Yes, it is fine, I get medication as usual, I use it, when it finishes I go back. I am grateful. Right now, I am a little better because I used to faint every time.

I: Okay, how?

P: Normally, when I feel bad, I don’t know what my brain does, I faint; when I go to the hospital that is when I regain consciousness and realize I am at the hospital. Because I have all types of Blood Pressure, I have low Blood Pressure and high Blood Pressure. I have them all.

I: Okay. And which would you rather, to receive treatment for your Blood Pressure at this CTC or to continue receiving treatment where you are getting it now?

P: Medication is medication. All I want is to get treatment and medication to reduce my blood pressure…

I: …Okay. So, if you were to get it here [Mwananyamala]…

P: …but even here I can come and get treatment as usual.

I: Have you ever asked if they have Blood Pressure services here?

P: Here?

I: Yes.

P: Honestly, I have never asked.

I: Okay. And you have never wanted to follow up?

P: yes, because normally I get medication there [Kairuki] that is it, I come here [Mwananyamala] with my other problem [HIV]. And I usually use insurance.

I: Okay. And what would you advise be done in order to get better Blood Pressure treatment here at Mwananyamala CTC?

P: If there is a clinic for it [Blood Pressure] here, and there is medication, I would come…

I: so as long as there us medication…

P: …yes. So that I can just use it.

I: Okay.

P: because the ones I get here would be for blood pressure and the ones I get there would be for blood pressure…the same thing. This is a big hospital and it has been our hospital for a while.

I: Okay. So, you have no problem going there and here?

P: It is better here where I get treatment [for HIV] if Blood pressure is also moved here it would be better.

I: Okay. Thank you Fatuma those were my questions, if you have anything to add regarding treatment of blood pressure here at Mwananyamala CTC…

P: I do not have any questions and nor do I have any problems.

I: Okay. Thank you.
