# Supplementary material for: Factors hindering integration of care for non-communicable diseases within HIV care services in Dar es Salaam, Tanzania: The perspectives of health workers and people living with HIV
Source: PLoS One. 2021 Aug 12;16(8):e0254436. doi: 10.1371/journal.pone.0254436 (PMC8360604; doi:10.1371/journal.pone.0254436)
Supplement: S4 File — (ZIP) [file pone.0254436.s004.zip › Transcripts PLHA/CTC4 02 docx.docx]

NCD STUDY: DIABETES

LOCATION: MWANANYAMALA

INTERVIWER: D K

PATIENT: 02

I: Hello (…)

P: Hello.

I: My name is Diana, I come from MDH, as I explained to you in short before we began…

P: Okay.

I: …I wanted to hear about your experience regarding diabetes services and treatments at this CTC…

P: Okay.

I: …before we start though, I would like you to tell me your full name, your age, your education level so that I can get a little about your background.

P: Okay. My name is (…)

I: and your age…

P: I am 52 years old.

I: Okay, and your education level?

P: Grade 7, at Morogoro.

I: Okay. And are you married?

P: Yes, I have a wife and children, and also Grandchildren.

I: Okay congratulations. And what work do you do?

P: I do business.

I: Business. Okay.

Do you receive Diabetes treatment at this CTC?

P: I have never received treatment here.

I: You do not receive it here?

P: No, but I do get [treatment] at Darajani in Manzese.

I: Manzese? There is a hospital there?

P: Yes.

I: So, you also get treated there.

P: yes, or sometimes I just go there to buy medicine.

I: Okay.

So why do you not get treatment for Diabetes here at Mwananyamala CTC?

P: I was not informed. For example, today, they passed around and asked [nurses asked patients who had NCD in order to get participants for study], and because I know I have this problem I stood up.

I: Okay, so you did not know that there was Diabetes treatment at this CTC at Mwananyamala?

P: I did not know.

I: Okay.

What eases the ability for you to receive treatment or medication for your diabetes outside of this CTC clinic?

P: Many times, when you decide to go get medication, I can just buy them because I still have the receipts from the previous medicine...

I: Okay. So, you do not have to go to the Doctor and get checked and get a prescription?

P: No.

I: So where do you usually buy medicine?

P: I can buy from a pharmacy, or Faruz Pharmacy because they are my neighbors…

I: Okay, a pharmacy that is near where you live?

P: Yes.

I: Okay. And what does not allow or causes challenges for you receiving treatment or getting medication for Diabetes outside of this CTC clinic?

P: There is no reason that would not allow me other than initiative.

I: Can you please explain that a little more, initiative in what way?

P: Initiative in terms of; lets us say I woke up today and I look for a center that is nearby, I go test my levels [diabetes] and see what the amount is, they give me medication and I may use it and when I finish it [medication] I may decide to go to the pharmacy and just buy more.

I: Okay. Are you satisfied with the services you get to treat diabetes at the clinic outside of this CTC or the place where you are being treated now?

P: No, you can have faith with the service, but one day you may start wondering why can I not find a clinic which is where I usually attend…

I: Okay. Now that is where my next question comes about; so that is like a yes, but if it were a no; what would you prefer, to get diabetes treatment here at the Mwananyamala CTC which you currently attend or to continue getting treatment anywhere else?

P: I would prefer to get it from the clinic…

I: This clinic?

P: Yes, because I would be monitored…

I: …okay a clinic which you are used to and get… [treatment]

P: …. Yes!

I: Okay. And what would you advise be done in order to get better Diabetes treatment here at CTC?

P: What would satisfy me, firstly I have become like a regular, since the year 2009 I have been coming to this clinic and I have not left, so even if I were to be linked [diabetes treatment] here, I would still be in my usual area…

I: Okay. And what would like to be done so that you are able to get treatment here?

P: Me?

I: Yes.

P: Mostly I would like in terms of registration to be registered…

I: Okay….

P: …Yes, at this clinic.

I: To be registered how? Diabetes??

P: Yes…

I: Okay.

P: I know once I have been registered I am now in a better position to receive treatment and to be examined more thoroughly.

I: And anything else?

P: There is nothing.

I: It is just about registration?

P: Yes.

I: Okay. Do you have anything else to add regarding Diabetes treatment at this CTC?

P: Honestly, I do not have any problem which is very big. The only assistance I need is to get medication which is given according to the situation.

I: Okay, thank you, those were my question.

P: Okay.
