# Supplementary material for: Factors hindering integration of care for non-communicable diseases within HIV care services in Dar es Salaam, Tanzania: The perspectives of health workers and people living with HIV
Source: PLoS One. 2021 Aug 12;16(8):e0254436. doi: 10.1371/journal.pone.0254436 (PMC8360604; doi:10.1371/journal.pone.0254436)
Supplement: S4 File — (ZIP) [file pone.0254436.s004.zip › Transcripts PLHA/CTC5 14.docx]

**IDI:** NCD STUDY FOR HIV PLWHA

**SITE:** AMANA

**INTERVIEWER**: L. L

**EDUCATION LEVEL:** PRIMARY SCHOOL

**JOB TITTLE:** BUSINESS

**MARITAL STATUS:** DIVORCED

**SEX:** FEMALE

**AGE:** 50YRS

**TYPE OF DISEASE**: HYPERTENSION

**I:** Kindly tell me, what facilitates or hinders access to care of NCDs within CTC and what can be done to improve it?

**Re**: Let me be just honest, I wouldn’t know because the availability of hypertension drugs in.. CTC. Drugs are available especially the ones for HIV. But for these other diseases they would also be available if they had proper arrangements. **[**I asked the patient to speak louder**]** If they had proper arrangements in their budget they could have their stock.

**I:** You mentioned about budget arrangement, please it explain well.

**Re:** I mean, we get these HIV drugs for free? (Yes)I think they would make it the same for hypertension disease else or they would be here or we would have gotten them here in this facility.

**I**: Do you get your treatment for hypertension from this CTC?

**Re:** I don’t get it here. I get it from there…My son works at Hindu Mandal after I had repeated chest tightness. At that time when I was sick I would go to Mbagala Zakhem and every time they checked me up my pressure was high. Every time they checked me up my pressure was high. So they told me if the condition is like that I go to a big hospital to check my heart condition and when I went there they noticed my blood pressure was high. They prescribed me drugs and I am now doing well. My heart is okay except for this blood pressure. When I started medication my blood pressure was very high but for now am grateful. I am okay, last time I checked my blood pressure it was…I have forgotten that paper but it was normal, it was one hundred and thirty something.

**I:** So your problem is pressure and not pressure and heart?

**Re**: Yes, I was told its just pressure.as it’s just the day before yesterday I had a chest tightness and pain. I went for heart test before I was told it doesn’t have any problem. My problem is just pressure.

**I:** So you are getting your treatment at Hindu Mandal?

**Re**: Yes

**I:** Just like you said you don’t get treatment here at Amana, kindly tell me why, how, who and when you were diagnosed with this disease?

**Re**: Mhh, pressure?

**I:** Yes.

Re: Generally it’s been three years now. Just like I told you initially I was diagnosed at Zakhem. Every time they checked me they found my blood pressure was really high. They told me with that kind of pressure I would have to go to a big hospital to check if I had problems with the heart. Why is it all the time we give you treatment it does not help you? So when I went to postal, at Hindu Mandal they prescribed drugs which has given me relief and am still taking them. I can’t remember the name but I have some of them with me here [Showing her drugs].These are the drugs I have been taking since I started treatment and they really help me.

**I:** Okay, before you mentioned the reason for you to go to Hindu Mandal was your Son (Eeh, if it wasn’t for my son I wouldn’t have gone there).Are you satisfied with their services that you wouldn’t want to move here which is nearer?

**Re**: It just depends on the availability of my drugs. For example if they can be available here like the way we get these ARVs for free and these ones are here for free, we can come here and there will be no need to go there. Like if I come here I get all my drugs and go home.

**I:** What are the things that make it easy for you to get your drugs to treat you hypertension outside this CTC?

**Re**: Sorry?

**[**I repeated the question**]**

**Re:** It makes it easy for me because of my son who works at Hindu Mandal as my treatment cost is deducted from his salary. It’s not like I have money, I don’t have money. Life is difficult. Like today I came from Luanga, I take my drugs and go back to Luanga. [Car’s reverse alarm noise].If I take them all together it becomes better.

**I:** As you said your son is the one paying for your drugs at Hindu Mandal (Yes).If they said he can pay and you get them from here, would it be also easier for you?

**Re**: I wouldn’t be able to pay because they deduct straight from his salary there. I am the one who will…I don’t think I would be able to pay.

**I:** So money would be a problem.

**Re:** Yes.

**I:** What are the things that make it hard for you to get your drugs to treat you hypertension outside this CTC?

**Re:** Pardon!

**[**I repeated the question**]**

**Re**: To be honest I have not encountered any problem becomes they offer me good services every when I visit. I think I have not understood the question, I don’t know it’s because am very hard to understand.

**I**: No, you’re with right the way you answered.

**Re**: For me to say the truth, when I go there they offer me good services.

**I**: What about time?

**Re:** It depends on the time you have reached there. You go early you will live early and if you go late you will live late.

**I**: So there are no challenges?

**Re**: No challenges at all.

**I:** Are you satisfied with the care for your hypertension which you get from the place where you are receiving treatment for now?

**Re**: I receive good care from both clinics if you reach there early like I said, if you are late you find the clinic is full. [Words are not clear]. Otherwise they serve me well and am really served well.

**I:** Are you talking now about Hindu Mandal where you get treatment for your hypertension?

**Re:** Even here at CTC.

**I**: I wanted to know more about Hindu Mandal as you receive treatment outside this CTC, So how do you see them? Are you satisfied with Hindu Mandal services?

**Re**: That why I told you am satisfied as I cannot afford to buy these drugs. Am sponsored by my son because he works there.

**I**: Okay, and what would you prefer? To receive treatment for NCD or to continue at the clinic where you are attending now?

**Re:** I ask you to make it easy for me to understand, that’s why I told you am hard to understand.

**I:** Okay, what do you prefer most where you are attending now?

**Re**: There is nothing I prefer apart from receiving good treatment, that’s what am grateful to God for.

**[**I asked a question outside the contents**]**

**I:** Okay, so what is your opinion about receiving all your treatments at the clinic where you are attending now?

**Re**: They should get us these drugs and make them for free. For example this one right now has been removed from health insurance coverage [Ahaa, this one called Telmisartan has been removed from insurance coverage?]Yes, but if they can be prescribed here either at a low cost or for free we will then be grateful.

**I:** Nothing else that should be done better?

**Re:** Yes, nothing unless there is something you can elaborate for me.

**I:** Thank you, we have reached the end of our interview.

**Re:** Thank you.
