# Supplementary material for: Factors hindering integration of care for non-communicable diseases within HIV care services in Dar es Salaam, Tanzania: The perspectives of health workers and people living with HIV
Source: PLoS One. 2021 Aug 12;16(8):e0254436. doi: 10.1371/journal.pone.0254436 (PMC8360604; doi:10.1371/journal.pone.0254436)
Supplement: S4 File — (ZIP) [file pone.0254436.s004.zip › Transcripts PLHA/CTC1 19 rtf.rtf]

IDI SINZA PARTICIPANT FEMALE II

Attending both clinic at Sinza hospital (Pressure & CTC)
Previously she was selling food but due to illness she stopped and current depend from children for financial support.
Started attending pressure clinic 2015.
Not married
65 years
Never been to school
Staying at Kimara

The woman delayed to be diagnosed with pressure and used to fall in the street and other places and lost most of his personal items. She complain on the loss of memory and lack financial support to able to receive medication on time. The one child that she most depend from faced some challenges at working place and he is currently at home. 

Interviewer: Welcome to our interview   
Respondent: Thank you
Interviewer: I would like to know when did you started treatment for pressure

Respondent: It was a long time I started the treatment at Mwananyamala then I was shifted to Sinza at 2015 (showed a card to interviewee)

Interviewer: How were you diagnosed that you have pressure

Respondent: I was living at Magomeni and was a food vendor then I was not feel okay and felt pain on the shoulder and breast then I was advised to go for testing at Mwananyamala then I was referred to Muhimbili after getting the results then I was told my heart expanded (moyo wangu umetanuka) then I started clinic Dr XXX wrote laboratory test that I should take, then I had goiter and was supposed to be operated (interruption someone entered in the room).

Interviewer: Can you tell how you receive treatment for pressure

Respondent: I attend clinic every month and I took my pills every morning, I did not take the pills today because after taking the pills I do not stay in one place, every time I go to washroom to urinate

Interviewer: How frequently are you getting your pills

Respondent: Telling the truth due to the economic situation there are times that I cannot get because among my children one of them is one who assist me and he currently moved back to the country from Mozambique he has some difficulties in his business therefore it also affect how I get my pills sometime I get three days dose sometimes I purchase half of the dose sometimes I purchase the whole dose that is my life

Interviewer: What are the simplest way for you to get your medications?

Respondent: My children arrange for paying for health insurance for me because they sometimes went and ask for the price for some of the medications and they found they are sold 50,000 and sometimes I do not have that money to purchase the pills after the follow up they found the health insurance are very expensive. It reached a time that I stay without any medication.

Interviewer: What are the things that you think they need to be done so that you will be able to get the pills on time? 

Respondent: I don't know because it has become difficult for the kids that support me to pay for my medications.

Interviewer: Do you have recommendations on how well the service can be provided

Respondent: If there could be an option that you can tell that I will be able to be assisted you can tell. My problem is medication. I can stay without taking the food at night but my problem is the pills.

If I miss a dose I feel serious pain that I cannot sleep, I will just sit at nigh and have more pain on the chest side and cough a lot like someone with asthma during the CORONA time I was not able even to touch my grandkids it was a strange situation it was through the hospital assessment then they found out the my heart enlarge (above the normal size).

Interviewer: Apart from lack of money what else do you thing it's a challenge for you to access the medication that you want.   

Respondent: Doctor if you could take me and stay with me so that you can help me not to miss my dose, my problem is medication and how to be assisted to get the pills what can I do I have no option it is you doctor to advise me on how to take my pills I cannot afford I have no any option I cannot support even my grandkids when they go to school until one of them decided to beg to other kids when she reach school, one day I was called at school because of that behavior and explain to teachers how hard economic the situation is.  

Interviewer: Are you satisfied with heart/pressure treatment that your given?

Respondent: The problem for this treatment is the lack of medication. I use both CTC medications and pressure medication and one of the doctor said am taking a big dose and he made some changes on medications and it helped me as the first combination were too heavy for the body.

Interviewer: How do you see the way you're taking your medications at two different places within the same facility (CTC and OPD) 

Respondent: My request if it's possible the two clinics can be merged together 

Interviewer: What do you think are the advantage if the two clinic will be merged together 

Respondent: I'm worried if it reached a day the two clinics that located separate, am supposed to attend all of them in one day, if both clinic will be on the same day how will I divide myself

Interviewer: How are you attending your clinics now?

Respondent: On the previous day I did not have medication I decided to come for the pressure clinic on the other side of the building then I came to CTC clinic also requesting brother XXX some pills then he gave me some pills to help me, this is because I lost things like hospital cards, certificates, even important things as I speak now I don't know where I put the X-ray that I took at this clinic I just have the one that I took at Mazense. When I put something I do not recall. 

Interviewer: Thank you very much, this is the end of our interview.
