# Supplementary material for: Factors hindering integration of care for non-communicable diseases within HIV care services in Dar es Salaam, Tanzania: The perspectives of health workers and people living with HIV
Source: PLoS One. 2021 Aug 12;16(8):e0254436. doi: 10.1371/journal.pone.0254436 (PMC8360604; doi:10.1371/journal.pone.0254436)
Supplement: S4 File — (ZIP) [file pone.0254436.s004.zip › Transcripts PLHA/CTC2 15 rtf.rtf]

IDI Temeke Male
Divorced
69+
Not doing anything for generating income
Completed standard IV
Living at Temeke
Attending both CTC and Pressure clinic at Temeke

The old man was sick and not well satisfied with the amount of service that he received, he was divorced because of the financial constrains that he also face in accessing the quality of health service he want too. He cannot see at night therefore he has to rush day hours to get the service and reach home early, due to age he has challenge with movement.

Interviewer: 	Welcome to our interview baba

Respondent: Thank you

Interviewer: You have told me that you attend both clinic here at Temeke CTC and pressure

Respondent: Yes, through social welfare

Interviewer: When did you start to attend pressure clinic

Respondent: It is a long time now 

Interviewer: What can you share with us regarding the pressure treatment that you receive?

Respondent: When I get the medication I feel better I can stay for three or two days without feeling dizzy but if I did not get medication and try to wake up I feel very unbalanced then I fall down, there is a time that I fall down because of that and I got this mark (he showed the interviewee a black mark on his face)

Interviewer: Why are you taking your pills trough social welfare?

Respondent: It a close place that I can go and access the pills in other places I cannot afford 

Interviewer: At the begging you have told me there are times that you miss your pills 

Respondent: Yes, I have to buy them

Interviewer: What are the main reasons that you are missing your pills? 

Respondent: It's because of the money, at the hospital they prescribe to us and tell us to go and purchase at the pharmacy if I could have money I could buy my medication every after two days.

Interviewer: What is the thing that you like most in the presser treatment that you receive now 

Respondent: It took a while since I arrived at the clinic and the time that I started receiving the treatment. I waited.

Interviewer: What are the things that you think need to be done to improve the service? 

Respondent: If they could provide us with the pills that we can use then we can be satisfied with the treatment. Its only service. 

Interviewer: Are you satisfied with the service that you're provided

Respondent: What can I do? Anything that you cannot access if you force you will be hatred 

Interviewer: What are the things that could make you more satisfied? 

Respondent: Its only medications for pressure. 
Interviewer: Is there anything that you would like to share with me regarding CTC treatment that you receive here 
Respondent: All is good

Interviewer: How about the treatment for pressure

Respondent: I have not receive that treatment at large I cannot say anything about it

Interviewer: Why you have not get pressure treatment for the large amount you wanted

Respondent: It is because of our face (he is old) some of the attendant need money due to the current situation and some they do not want money they just want to help others in life.

Interviewer: Thank you baba.
