# Supplementary material for: Factors hindering integration of care for non-communicable diseases within HIV care services in Dar es Salaam, Tanzania: The perspectives of health workers and people living with HIV
Source: PLoS One. 2021 Aug 12;16(8):e0254436. doi: 10.1371/journal.pone.0254436 (PMC8360604; doi:10.1371/journal.pone.0254436)
Supplement: S4 File — (ZIP) [file pone.0254436.s004.zip › Transcripts PLHA/CTC2 22 rtf.rtf]

IDI NCD TEMEKE 
19.08.2020
FEMALE
ATTENDING PRESSURE AND DIABETIC CLINIC AT HINDU MANDA CTC CLINIC AT TEMEKE.
52YEAYS
SMALL BUSSINES UBUYU/KARANGA
STANDARD SEVEN 
NOT IN RELATIONSHIP

The patient was very polite, talking at a low notation. Not happy with all the suffering she is going through and currently not having a close person to share things with.
  

Interviewer: Welcome to our interview. Can you please tell us when did you started the treatment on pressure and diabetic?

Respondent: It was June, when I attend CTC clinic here, I was fat and they were worried about me after the treatment they sent me for another checkup then they invent that pressure and diabetic but for the diabetic I was taking the precautions it was not that serious compared to pressure. 

Respondent:  After they found out that you have pressure and diabetic what happened, I found it as a strange thing because I have never experience such situation.

Interviewer: When did you start treatment for pressure and diabetic?

Respondent: On the following month they gave me like one week

Interviewer: What happened then you went to Hindu Mandal for the treatment

Respondent: I received a call from Temeke that instruct me to go Hindu Mandal and I did not asked why I should go to Hindu Mandal

Interviewer: How did you see the service you receive for the diabetic and pressure treatment 

Respondent: The service provided is very good

Interviewer: When did you start treatment for diabetic

Respondent: On 3rd July 

Interviewer: Have you ever asked for diabetic and pressure treatment here at Temeke?

Respondent: No, I have never asked 

Interviewer: what kind of the body sings that when you see/notice in your body that you feel  you need treatment starting with diabetic 

Respondent: The main issue is pressure on diabetic the issue is not too bad

Interviewer: How do you feel in your body that led you to think that you need treatment for pressure?

Respondent: I used to feel serious head pain at the middle of the head sometime I feel weak and I don't know why.

Interviewer: What are you doing after feeling that 

Respondent: I drink plenty water and eat a lot of cucumber 

Interviewer: How do you take your medications?

Respondent: I take one and a half in the morning 
Interviewer: Have you ever been told by your doctor when you feel that situation what are you supposed to do 

Respondent: No I have not be told by the doctor

Interviewer: Have you ever asked the doctor

Respondent: No, I have not 

Interviewer: What are the challenges that hinders you to receive treatment for the pressure that you wish you could get 

Respondent: I wish I could be able to take the taste every time when am not feeling well, take the pressure assessment on time. I wish I could have measuring machine.

Interviewer: Another challenge

Respondent: I cannot run my business throughout the time there is time that I need to go for my business and I start feeling sick.

Interviewer: Is there any way that you have tried to work on to be able to have a pressure testing machine that you wish to have

Respondent: No, I have not 

Interviewer: Why did you like to proceed attending your clinic at Hindu Mandal have ever thought of another clinic

Respondent: No I have not, after receiving a phone and because I cannot reject and I know nothing, I follow instructions that am given.

Interviewer: Who did you called you?

Respondent: I was called by sister XXX and said I have to go to Hindu Mandal and I was not aware of the place and she gave me directions.
Interviewer: What are the challenges that you met with in the process of attending your clinic

Respondent: Transport until I arrive at the clinic, I get out of the house at 04am and arrive at the clinic at 6am.
Interviewer: Are you satisfied with the service that you receive

Respondent: I'm satisfied, they are welcoming and the service that they provide are good.

Interviewer: What are the things that you're happy with the treatment that you receive at Hindu Mandal

Respondent: I do not pay for anything for the treatment. I cannot afford to pay for the treatment at that hospital but I took all the test without paying any money I don't know why because at that hospital you're supposed to pay cash or use health insurance and they have not asked me, I just see I get treated and given pills then I left.

Interviewer: What do you think needs to be improved so that the treatment that you receive could be more better? 

Respondent: They should only proceed to treat us well, they should also give us pills for pressure and diabetic. I have not yet get challenge on medication so far they should proceed helping us.

Interviewer: Thank you very much this the end of our interview.

Respondent: Thank you.


 
