# Supplementary material for: Factors hindering integration of care for non-communicable diseases within HIV care services in Dar es Salaam, Tanzania: The perspectives of health workers and people living with HIV
Source: PLoS One. 2021 Aug 12;16(8):e0254436. doi: 10.1371/journal.pone.0254436 (PMC8360604; doi:10.1371/journal.pone.0254436)
Supplement: S4 File — (ZIP) [file pone.0254436.s004.zip › Transcripts PLHA/CTC1 18 rtf.rtf]

 
IDI SINZA

Age: 70 yrs.
Female
Never been to school
Not doing anything for generating income
Pressure patient attending clinic at Mwananyamala and CTC clinic at Sinza.
August 2020.

Bibi suffered from serious pressure for a long period of time (more than 20 years) and there was a time that she was totally unconcise stayed at ICU for a week, family members were worried that she died. After a week she woke up.
She has poor adherence on pressure treatment for almost six month, she was supposed to move to Sinza hospital where she attend CTC clinic but unfortunately she cannot pay ten thousand shillings as a registration fee that will also allow her to register and open a file.

Interviewer: Welcome to our interview bibi xx, how are you 

Respondent: Just like that, because am breathing I thank God

Interviewer: When did you start to suffer from pressure?

Respondent: It was a long time now (she was not able to recall the exactly date)

Interviewer: What was the source of you getting pressure?

Respondent: I was just normal, my late husband was the one who was having pressure. My husband was working as manager at XX so we were getting treated at their hospital therefore one day I was feeling bad and thought was a fever then I decided to go the hospital they asked me how I felt and they told me I have a high blood pressure it a long time now.

Interviewer: Can you tell us about the health service that you're receiving 

Respondent: What can I do, am given pills and I take but I have not recover because the pressure was going high up to 200-220-230 up to 250 then they told me that this pressure will lead to heart problem and it was true after some time I felt more weight on one side then I went for checkup and I was told I HAVE A BIG HEART, THE HEART EXPAND, up to now, I'm attending both pressure and heart clinic. 

Interviewer: Are you receiving all the treatment service that you need?

Respondent: They give me pills a bit and I took them that how it is

Interviewer: Have you ever missed a dose

Respondent: I was told to take pills everyday but they need me to shift to Sinza were I attend another clinic for HIV and I was told to pay ten thousand at Sinza so that they could register me and open the file for my pressure clinic, I told them I do not have money and the child that was helping me died.

Interviewer: Therefore you're not take medication for pressure now

Respondent: Yes, I did not take any almost five month now, sometime when I go to Mwananyamala they do not accept me and ask me to go back to Sinza were I should attend both clinic for HIV and pressure now when I go to the government hospital they give me LUXES that help me to urinate and keep telling me to go to my clinic.

Interviewer: You're taking LUXES?

Respondent: I take only LUXES they help me to urinate but they do not help me anything because they used to give me even four different types of LUXES when I was at my clinic.

It almost five to six month now if I come to Sinza they send me back to Mwananyamala when I go to Mwananyamala they send me again to Sinza, I just left this to God if it's the last chance to survive or I will die then its fine.

Interviewer: What is the main reason that you're missing the required clinic?

Respondent: They asked me to pay ten thousands so that they could open a file to me and I told them I do not have the money, I cannot even feed myself that is the reason.

Interviewer: What do you think need to be done so that the service that you receive could be more improved?

Respondent: I don't know, I just listen to you

Interviewer: Do you have any opinion that could make the service more improved?

Respondent: My opinion I request you to help me as your mother and grandmother I cannot afford am just living, I depend on God he is the who know my life, I do not have ability the one child that I was depend for he died and the one that remain he does not work, it's hard for us to afford even the meal.

After the death of my son who was supporting me it gave a hard time and I tried to console myself otherwise it could be worse due the pressure that am suffering from even at the time that he was admitted at Mwananyamala the pressure was high throughout the time I was admitted and we were on the same room, I got injected and we were all in the same ward. It was hard even for the relatives they lost hope that I could survive.

Interviewer: On the side of HIV and pressure medication do you have any recommendations on how better the situation could be improved 

Respondent: I have no comment, I was told if I could not take the pills things will be worse and God helped that you serve as for free God will bless those who give and help us your serving us freely and health care providers are helping us we are very thankful.
