# Supplementary material for: Factors hindering integration of care for non-communicable diseases within HIV care services in Dar es Salaam, Tanzania: The perspectives of health workers and people living with HIV
Source: PLoS One. 2021 Aug 12;16(8):e0254436. doi: 10.1371/journal.pone.0254436 (PMC8360604; doi:10.1371/journal.pone.0254436)
Supplement: S4 File — (ZIP) [file pone.0254436.s004.zip › Transcripts PLHA/CTC5 07.docx]

NCD STUDY: HYPERTENSION AND DIABETES

LOCATION: MWANANYAMALA

INTERVIWER: D K

PATIENT: 07

I: Before we begin (…), I wanted to get a little bit of information about yourself; you age, education, weather you are married or not and the work you do. But first I would like to ask you to tell me your full name.

P: My name is (…)

I: Okay. And how old are you (…)?

P: I am currently 39 years old...

I: Okay. And what is your education level?

P: I ended at Grade 7.

I: Okay, and are you married?

P: Yes, I am married.

I: And what work do you do?

P: The work I do entails helping a woman who cooks street food, and from there I get about tshs. 2000 – 3000...

I: Okay. In the beginning [before beginning the interview] you had told me that you have Diabetes and Blood pressure…

P: Yes.

I: …and do you get treatment for Diabetes and Blood pressure at Mwananyamala clinic?

P: Yes.

I: The place where you go collect your ARVs?

P: Yes.

I: Okay. The same exact place where you collect your ARVs?

P: No, room number 20 is the one for Diabetes…

I: Okay.

So, it is not at the CTC??

P: yes, not the place for HIV, but it is at Mwananyamala Hospital….

I: Okay…

P: room number 20…

I: Okay.

And do you get your medication [Diabetes] from the CTC or at room number 20? The Diabetes and Blood pressure medication…

P: No.

I have never gotten Diabetes medication because they tell us to go buy them, they write a prescription and tell us to go buy.

I: Okay, so where do you go buy them [Diabetes medication]?

P: At Pharmacies…

I: Okay.

P: they write a prescription and we go show it [at the pharmacy] and then buy …

I: Okay. And the blood pressure medication?

P: I have not yet started using the blood pressure medication...

I: You have not started using them?

P: Yes.

I: And what is your opinion about getting all the treatments at the same time at your ARV clinic? How would you feel about that?

P: We would be very happy; we get stressed because you may find you have the same date for both, so you go from HIV and then have to go Diabetes and it is all on the same day but you find you are unable to attend both because they can tell you the time is up so go away [the time for the second clinic has concluded while she was in the other one]…

I: Okay. And what things discourage or challenge your ability to get your Diabetes medication?

P: I get challenges because buying them is very expensive, and sometimes I may not get them and end up staying a whole week without injecting; because I do not have money and these days there are no longer free…

I: Okay. So, there was a time when they were free?

P: Yes. I have gotten them for free once when I was starting. Because they diagnosed me with Diabetes after my finger started hurting, I was admitted to Mwananyamala and my finger was cut off; now the first day after getting out of the ward, I went there [room 20] and they gave me two small bottles for mixing that medication and injecting it; now since I took it them that day until today I have not taken them again…

I: Okay.

Are you satisfied with the diabetes health services that you receive?

P: (sighs) no. we are not satisfied. Because we can only say we are satisfied if we were receiving medication, but we do not get medication; we are not satisfied because some of us do not have the ability to buy the medication, you have to work as being a street cook and save and save and save until you get to enough for one bottle which is 17000-18000; and I need to be injected with two [bottles]! So sometimes you are not able to [save enough money] and you miss using medication…

I: Okay, that is true…

P: Yes.

I: And what do you advise be done so that you can receive better treatment for Diabetes at Mwananyamala ARV clinic?

P: If you could assist us to be able to get at the same time. When the medication finishes, we tell you and if possible, we also get Diabetes medicine in the same way that we get ARV. It would be much better if we got that medication [Diabetes] in the same way that we get those medication [ARV] at the same time. We would really appreciate it, you would have really helped us.

I: Okay.

Thank you, those were all my questions. Have a good day.

P: Okay. Thank you.
