# Supplementary material for: Factors hindering integration of care for non-communicable diseases within HIV care services in Dar es Salaam, Tanzania: The perspectives of health workers and people living with HIV
Source: PLoS One. 2021 Aug 12;16(8):e0254436. doi: 10.1371/journal.pone.0254436 (PMC8360604; doi:10.1371/journal.pone.0254436)
Supplement: S4 File — (ZIP) [file pone.0254436.s004.zip › Transcripts PLHA/CTC5 11.docx]

**IDI:** NCD STUDY FOR PLWHA

**SITE:** AMANA REGIONAL REFFERAL HOSPITAL

**INTERVIEWER**: L. L

**EDUCATION LEVEL:** PUBLIC SERVICE COLLEGE

**JOB TITTLE:** BUSINESS

**MARITAL STATUS:** WIDOW

**SEX:** FEMALE

**AGE:** 59 YRS

**TYPE OF DISEASE**: HYPERTENSION

**I**: Hello mama,

**Re**: Hello.

**I:** Kindly tell me, what facilitates or hinders access to care of NCDs within CTC and what can be done to improve it?

**Re**: Nothing hinders, it’s just that we patients ourselves are not open with how we feel with our bodies. We do not visit the hospitals early.

**I:** Why do you think people do not go to the hospital early?

**Re**: We sometimes feel like it’s just a normal state when having headaches.

**I:** Is it what hinders access to treatment?

**Re**: There is treatment, it’s just us who do not go to the treatment centres. There is treatment for heart problems in all government facilities.

**I**: So you patients yourselves are the only hindrance to access to care.

**Re**: Yes.

**I**: Okay, so what do you think should be done?

**Re:** Motivate people. For example yesterday I went to Amana, I left Temeke and went to Amana. I found that they were motivating patients as there was free hypertension checking, blood sugar checking and cervical cancer screening. So it is simply motivation and people to get more education.

**I**: Do you get your treatment for hypertension from this CTC?

**Re**: No, I get it at Mwananyamala.

**I**: Mwananyamala?

**Re**: Yes.

**I:** Okay

**Re:** [Her words are not heard clearly] Mwananyamala.

**I:** Okay, you mentioned that you get your treatment at Mwananyama, isn’t it? And not here at CTC. (Yes) Why have you decide to go to Mwananyamala ?

**Re:** I was admitted and so they decided I should start my clinic there.

I: What were you admitted for?

Re: Hypertension.

I: Where was it?

Re: Mloganzila, the one affiliated to Muhimbili.

I: Then what happened until you were admitted at Mloganzila and started your hypertension clinic at Mwananyamala?

Re: They asked me to choose the closest clinic. [Words not clear].

**I**: Why Mwananyamala?

**Re**: It is easy for me.

**I:** Do you leave near Mwananyamala?

**Re**: No [Laughs].

**I:** So it is Mloganzila where they told you to choose? [Between Mloganzila and Mwananyamala where I came from].When were you diagnosed?

**Re**: It was in December, last year but one.

**I**: Okay, what are the things that make it easy for you to get your drugs to treat hypertension or to get treatments outside this CTC?

**Re**: At Mwananyamala we buy the drugs. [She keeps quite a bit].We buy them except for those with exception and health insurance, but for me I have to buy them.

**I:** So nothing makes it easy for you to get your hypertension treatment there?

**Re**: Nothing at all.

**I**: What are the things that make it hard for you to get your drugs to treat hypertension outside this CTC?

**Re**: [Stays quite]

**I:** Did you understand the question?

**Re:** No.

**I:** I mean, what brings challenge for you to get your hypertension drugs or treatment outside this CTC?

**Re**: There is no challenge.

**I:** Why saying so?

**Re**: But where is CTC?

**I:** CTC is here where you get your ARVs services.

**Re**: But they are also at Mwananyamala?

**I:** Eeeh.

**Re**: There are no challenges.

[TV sound heard]

**I:** Nothing gives you challenges (Nothing) in getting these hypertension services?

**Re**: Not at all.

**I:** Okay. Are you satisfied with the care for your hypertension which you get from the clinic you are receiving treatment for now?

**Re**: Yes, It’s just the drugs that cost me.

**I**: What makes you say you are satisfied?

**Re**: Just good services, they check me up, ask questions and advise me on what food I should eat and about doing exercises.

**I:** So there are no challenges at all?

**Re:** The challenge is in drugs.

**I:** Drugs as in buying or availability.

**Re**: Buying, I mean money. Since am alone and the children depend on me for food, and hypertension drugs are expensive.

**I:** What would you prefer? To receive treatment for NCD at this CTC or to continue at the clinic outside this CTC where you re attending now?

**Re**: Only if I can get easiness for my treatment. If the drugs will be for free I can then be transferred to this clinic.

**I:** If the drugs will not be for free won’t you like to be transferred to this clinic?

**Re**: I will keep going to Mwananyamala.

**I:** Don’t you see signs of relief by getting your drugs here instead of going too far from where you also get your ARVs.

**Re**: Am already so much used to Mwananyamala, its far but am used to it.

[I asked a question out of the study content]

**I:** We have reached the end of our interview and allow me to wish you a good afternoon.

**Re**: Okay.

**I:** What do you advise to be done so that you will receive a better care for your hypertension at this CTC?

**Re**: On the hypertension side …You can stay in queue for a very long time waiting for the doctor. In the end you don’t see the doctor. They just prescribe you then you leave. But your aim was to see the doctor. So they need to add more hypertension doctors and not one doctor to treat this, treat that and at the same time treat hypertensive patients. There should be hypertension specialists that when the patient comes to the clinic they are sure about finding the doctor at the clinic.

**I**: Thank you, we have reached the end of our interview.

**Re**: Thank you.
