# Supplementary material for: Factors hindering integration of care for non-communicable diseases within HIV care services in Dar es Salaam, Tanzania: The perspectives of health workers and people living with HIV
Source: PLoS One. 2021 Aug 12;16(8):e0254436. doi: 10.1371/journal.pone.0254436 (PMC8360604; doi:10.1371/journal.pone.0254436)
Supplement: S4 File — (ZIP) [file pone.0254436.s004.zip › Transcripts PLHA/CTC5 06.docx]

NCD STUDY: DIABETES

LOCATION: MWANANYAMALA

INTERVIWER: D K

PATIENT: 06

I: Hello, my name is Diana, I am from MDH. I would like to ask you a few questions regarding non-communicable diseases and their services here at the Mwananyamala CTC.

P: Okay. My name is (…), I am 45 years old…

I: Okay. Are you married?

P: Yes, I am married.

I: Okay, and your education level is up to…

P: I ended at Grade 7.

I: Grade 7. And what work do you do?

P: I used to be a driver but after getting this disease I stopped and currently I am just doing odd jobs.

I: Okay. So, you normally come here to this CTC to get treatment for HIV and AIDS…

P: Yes, and Diabetes…

I: …and Diabetes. So, do you receive Diabetes treatment here at this CTC?

P: Honestly, I come here for tests [for Diabetes] …

I: You come to test for Diabetes?

P: Yes, but I get my medication from a wholesale shop in Kariakoo at Heko.

I: Okay. So, you do not get your medication from here?

P: In the beginning I used to get them [diabetes medication] from here, but after realizing it was expensive, I started getting them from Heko at the whole sale shop.

I: How long ago were you getting the medication from here?

P: About three years back.

I: Okay. So, the prices there [Kariakoo, Heko] are more affordable than here?

P: Yes, it is more affordable.

I: Okay. And what are your opinions about getting all the services combined here at your clinic in mwananyamala?

P: My opinion is that it would be easier for us we were provided with all the medication here, it would be much better.

I: Okay. What do you think would encourage or make it easier for you to get your medication and treatment for diabetes here at your clinic?

P: I have not understood the questions.

I: I mean, for example at the moment you do not get the medication here, and also; do you receive treatment for diabetes here?

P: I get tested here…

I: you get tested here at the CTC or somewhere else?

P: there in the middle [pointing to another building] …

I: at OPD?

P: yes, I think it’s called OPD.

I: Okay, now what do you think would make it easier for you to receive everything here? What would help?

P: Honestly, I would request that in the government’s plans, if they could help us get everything here at an affordable price, that would be much better.

I: Okay. And in your opinion are you satisfied with the Diabetes health services you receive here at the CTC.

P: I am very satisfied.

I: Very satisfied. Can you explain a little more about this?

P: First, the doctors really care about us; and testing is very cheap; they test for Pressure, Diabetes, Weight; all for just Tsh. 1000.

I: They do all this testing here at this CTC?

P: Yes.

I: Okay. But medication is…

P: Medication is the problem. There [Heko] one box of 100 pills I usually get for Tsh. 4000, Metaphomine I get for Tsh. 5000; here [Mwanyamala] it was a little bit more expensive.

I: Okay. And what would you suggest be done so that you receive better treatment for Diabetes here at this CTC?

P: If they reduced the price of treatment.

I: They reduce the price of treatment?

P: Medication. When it comes to tests they are fine.

I: Okay. So, the issue is concerning the medication?

P: Yes, Medication.

I: Okay, I have no more questions. If you have anything more to add concerning Diabetes treatment here at the CTC, or any last thoughts….

P: I would just like to ask the government to reduce the cost of the medication, and if it were possible, if they could also reduce cost on insurance…

I: okay…

P: …because yesterday I was at Mawasiliano, the insurance is high; and currently I honestly do not have money…I work odd jobs, so I may go work today, then stay home for two days and not work, then work again; so, money has become very hard to come by…

I: okay…

P: …I used to be a driver but right now my eyes cannot see well so I cannot work.

I: And another thing, are you satisfied that when you have to get treatment for Diabetes you go somewhere else instead of getting the treatment here?

P: I come here for tests…

I: So, they test here and provide treatment here?

P: In the beginning they used to have treatment here…

I: Okay.

P: …but after realizing the prices of medication were a little high, that is why I decided to go get medication at Kariakoo…

I: Okay.

P: …. but all the tests I do them here.

I: Okay. Thank you. I do not have any more questions…

P: …how much is insurance?

I: Insurance??

P: Insurance for adults?

I: I am not sure…. But thank you, I am not with my questions.
