# Supplementary material for: Factors hindering integration of care for non-communicable diseases within HIV care services in Dar es Salaam, Tanzania: The perspectives of health workers and people living with HIV
Source: PLoS One. 2021 Aug 12;16(8):e0254436. doi: 10.1371/journal.pone.0254436 (PMC8360604; doi:10.1371/journal.pone.0254436)
Supplement: Attachment — Submitted filename: Response to Reviewers.docx [file pone.0254436.s005.docx]

Tausi S. Haruna

Dar es Salaam

Tanzania

19 May 2021

[Email.tausi.haruna@bn.hkmu.ac.tz/](mailto:Email.tausi.haruna@bn.hkmu.ac.tz/)

tausiharuna2015@gmail.com

Tareq Mukattash
Academic Editor
PLOS ONE

Dear Academic Editor

**RE: RE-SUBMISSION OF A REVISED MANUSCRIPT**

I am kindly requesting a resubmission of a revised manuscript titled **“Factors Hindering Integration of Care for Non-Communicable Diseases within HIV care services in Dar es Salaam, Tanzania; The Perspectives of Health workers and People Living with HIV”** Id <https://orcid.org/0000-008-4117-3576>.

Thank you so much for your valuable time and useful contributions. I appreciate the comments and inputs given by both reviewers and yourself since they will definitely improve our manuscript.

Please find in the next section the detailed responses addressing reviewer’s comments for your perusal and further considerations

Sincerely yours

Ms. Tausi S. Haruna

| S/N | REVIEWERS | COMMENTS | RESPONSE | PAGE NO. |
| --- | --- | --- | --- | --- |
| 1. | **Journal Requirements:** | 1. Please ensure that your manuscript meets PLOS ONE's style requirements, including those for file naming. The PLOS ONE style templates can be found at  https://journals.plos.org/plosone/s/file?id=wjVg/PLOSOne_formatting_sample_main_body.pdf and  [https://journals.plos.org/plosone/s/file?id=ba62/PLOSOne_formatting_sample_title_authors_affiliations.pdf](about:blank) | I have visited the link for guidance and now files meet the requirements of the PLOS ONE’s style |  |
| 2. |  | **When reporting the results of qualitative research, we suggest consulting the COREQ guidelines: http://intqhc.oxfordjournals.org/content/19/6/349. In this case, please consider including more information on the number of interviewers, their training and characteristics; and please provide the interview guide used.** | **Research assistants were trained.** **The research assistants had a mix of pre-graduate diplomas in health and social science para professional field (n=3) postgraduate degree in Public health (n=1) and undergraduate degrees in the social sciences or Law (n=2) fields. All RAs also were up to date on research ethics certification (FHI360 certification course). Find attached interview guides in the supplementary files** | 7 |
| 3. |  | In the Methods section, please provide additional information regarding the interview guide development process, including the theories or frameworks which were employed. | **Information regarding development of interview guide has been included including framework** | 5-6 |
| 4. |  | Please include in your Methods section (or in Supplementary Information files) the participating hospitals/institutions. | **We have added…five major CTC namely, Mwananyamala**  **Temeke Regional Referral Hospital**  **Sinza Health Centre**  **Amana regional Referral Hospital**  **Vijibweni health Centre**  **Because this was a small study, we have decided not to include names of hospitals in the manuscript to protect DHCP and CTC managers but I have included permission letters for data collection in the Supplementary files** | See online submission |
| 5. |  | **Observational checklist at CTC on NCD issues**  Please provide the observational checklist as Supporting File. | **Attached as Supporting Files** |  |
| 6. |  | 3. We note you have included a table to which you do not refer in the text of your manuscript. Please ensure that you refer to Table 1 and 2 in your text; if accepted, production will need this reference to link the reader to the Table. | **Thank you**  **We have indicated the Tables in the document (Table 1 is for example of the analysis process of the main theme, categories and quotes, Table 2 Demographics data for PHLA and Table 3 Socio-demographics data for DHCP and CTC managers** | 8, 10 &11 |
| 7. | **Reviewer's Responses to Questions**  **Comments to the Author** | 1. Is the manuscript technically sound, and do the data support the conclusions?  The manuscript must describe a technically sound piece of scientific research with data that supports the conclusions. Experiments must have been conducted rigorously, with appropriate controls, replication, and sample sizes. The conclusions must be drawn appropriately based on the data presented.  Reviewer #1: No  Reviewer #2: Yes | **Thank you for our comment, we have now improved more on conclusion to make it appropriately based on the data presented in the manuscript See page**  (Thank you for the comment) | 24 |
| 8. |  | 2. Has the statistical analysis been performed appropriately and rigorously?  Reviewer #1: N/A  Reviewer #2: N/A | N/A | N/A |
| 9. |  | 3. Have the authors made all data underlying the findings in their manuscript fully available?  The [PLOS Data policy](http://www.plosone.org/static/policies.action#sharing) requires authors to make all data underlying the findings described in their manuscript fully available without restriction, with rare exception (please refer to the Data Availability Statement in the manuscript PDF file). The data should be provided as part of the manuscript or its supporting information, or deposited to a public repository. For example, in addition to summary statistics, the data points behind means, medians and variance measures should be available. If there are restrictions on publicly sharing data—e.g. participant privacy or use of data from a third party—those must be specified.  Reviewer #1: No  Reviewer #2: No | **All interviews transcripts and checklists have been provided in the supplementary files** | See online submission |
| 10. |  | 4. Is the manuscript presented in an intelligible fashion and written in standard English?  PLOS ONE does not copyedit accepted manuscripts, so the language in submitted articles must be clear, correct, and unambiguous. Any typographical or grammatical errors should be corrected at revision, so please note any specific errors here.  Reviewer #1: No  Reviewer #2: Yes | **Thank you for the comment, the manuscript now has been reviewed with professional language reviewer and grammatical errors have been checked and corrected) See page 1 to the end**  (Thank you for the compliment) | 1-27 |
| 11. | **Reviewer #1** | Reviewer #1: Thanks for considering PLOS ONE for your submission. I have noted points that I would like to share about your submitted work: 1. The submission contains many writing mistakes (typos, sentence structure issues e.g. run on sentences) (I recommend using English writing aid tools), consistency would help (checklist was written in many different ways). | **Thank you for your comment, we have now sent the work to language reviewer and now the typos, sentence structures and use of English aid writing tools have been used to support writing) See page 1 to the end** | 1-27 |
| 12. |  | 2.The short title is still long I recommend making it shorter. | **Thank you for the comment, we have now made a shorter title and make it shorter.** | See online submission |
| 13. |  | 3. Key words need to be revised and modified to facilitate the chance of locating your work if it gets published | **Thank you for the comment. We have now modified and revised the key words and now it reads Integration, Non-Communicable Disease, Qualitative, Tanzania, PLHA, Clinic and Treatment Centre, hypertension, diabetes** | See online submission |
| 14. |  | 4. I do not agree with including too many subheadings/subtitles as you have done with every section in introduction, try to provide coherent paragraphs, instead. | **Thank you for the comment we have now reduced the subtitles in every section and provided coherent paragraphs. The retained subtitles are for helping a reader to follow through See pages 1 to end** | 1-27 |
| 15. |  | 5. Providing numbers for lines would have made my job and yours easier to track my comments. | **(Thank you for the comment, we have now inserted line numbers to make our job easier. See page 1 to the end)** | 1-27 in the file with tract changes |
| 16. |  | 6. The second sentence in third paragraph in the introduction needs a reference | **Reference inserted. See Page 3-4** | 3-4 |
| 17. |  | 7. The ART abbreviation was not defined at first mention | **Thank you for the comment, we have now defined the ART abbreviation when first mentioned. See page 4)** | 4 |
| 18. |  | 8. Regarding study design, I did not find the primary assumptions from authors. I think this would raise the issue of personal bias while producing useful data from study participants, which may lead to biased hypothesis representing the phenomenon of interest. | **Thank you for the comment, we have now added a sentence…We prepared In-depth interview guides based on current literature on the recognition and integration of care for NCDs within CTC and trustworthiness section as an assumption to show authenticity of the design and quality of data. See page 10** | 5 & 9 |
| 19. |  | 9. I think some of your eligibility and exclusion criteria are redundant. | **Removed…in the targeted 5 district referral hospital**  **Removed....and facility/ CTC heads also known as CTC in charge.**  **Removed… who were not receiving ART**  **Deleted or did not provide signed informed consent as well as PLHA newly enrolled to**  **Deleted. those not directly involved with caring for PLHA and newly employed**  **We have merged this section into Study setting as per COREQ checklist** | 6 |
| 20. |  | 10. Both sections of sampling approach and study settings include same information (redundant). | **Thank you for the comment we have now removed sampling approach section and we have recasted study settings section to be more focus and avoid redundancy. See Page 6-7)** | 6-7 |
| 21. |  | 11. I recommend that you include further details about the development of the guide of semi structured interviews. | **Thank you for the comment, we have now added section data collection tools and we have included information on the development of the interview guide Refer page 5-6** | 5-6 |
| 22. |  | 12. Data analysis section includes some of the results, and it lacks the inductive content analysis process of analysis. More details describing the analysis is needed to show the link between extracted data and your reported results. | **Thank you for the comment, we have now recasted the data analysis section and put more description of analysis showing the link between the extracted data analysis and reported results) See page 8** | 8 |
| 23. |  | 13. I recommend that you include further details about the open coding/grouping steps and category creation and abstraction processes | **(Thank you for the comment, we have now recasted the data analysis section and put more description of analysis showing the link between the extracted data and analysis and reported results and put example of analysis See Table 1) See page 8 where the changes of analysis section has been done** | 8 |
| 24. |  | 14. I would like to see better organization by providing a conceptual model with your identified categories to give a meaningful sense of describing the selected phenomenon to increase readers understanding and help in generating knowledge. | **Conceptual model has been included**  **We have uploaded as supplementary file** | Figure 1 uploaded |
| 25. |  | 15. I would suggest to provide names or headings of higher order categories given your driven content characteristic words. | **(We have decided to provide themes which is more of abstraction level from the analysis we therefore suggest for maintaining those headings)** | 12-20 |
| 26. |  | 16. Providing Tables/figures including well labeled sub-categories and generic categories and main categories would be helpful | **We have formulated a table showing some of the theme and categories (Refer Table 1) and figure od summary of results** | 8 &12 |
| 27. | **Reviewer #2** | Overall, the paper is well written however, it a bit lengthy and both the introduction and the methods can be reduced. | **We have reduced some of the repeating quotes and merged some of the subtitle**  **We now have 27 pages from 35 pages** | 1-27 |
| 28. |  | - I enjoyed how the authors narrated the quotes in the results, however, I cannot see the benefit from table 3. It would be easier to limit the examples on themes in one place. I prefer to keep them in the results section and take out table 3. | **We have removed Table 3 in the results section.** | 9 |
| 29. |  | i recommend to move Onsite checklist-based observation findings to the prior to the results of thematic analysis. | **Thank you for your comment**  **We have now moved Onsite checklist-based observation findings before IDIs results** | 9 &10 |
| 30. |  | in table 1: I am not sure if you are meant to say "hypertension" instead of "hypotension" | **The word hypotension has been changed to hypertension. Please check Table 2** | 10 |
| 31. |  | in table 1: please check the (%) they do not add up for example:  Male 4(14.8%) Female 23(85.1%)  total is not 100%  same for Business 16(59.2%) Employed 1(3.7%) Unemployed 10(37.0%) | **Checked and corrected**  **Male 4(14.8%) Female 23(85.2%)**  **Business 16(59.3%) Employed 1(3.7%) Unemployed 10(37.0%)** | 10 |
| 32. |  | in table 1: ulcers! what kind of ulcers? | **Ulcers represent all types of ulcers** | 10 |
| 33. |  | I am not sure if table 4 is needed, the summarized results in the Onsite checklist-based observation findings in the results section can be sufficient. | **We have removed table 4 (Refer page** | 1-27 |
| 34. |  | Limitations: there are more limitations to be discussed related to the use of qualitative analyses. | **Thank you for this comment**  **We have now added another limitation related to study setting...** **Conducting IDs interviews at the clinic settings, where the participants receive their care/services may have also influenced them towards giving socially acceptable answers to the interview questions. The researchers mitigated this bias by asking open-ended questions, so that to make participants respond in a way that is not socially desirable.** | 23 & 24 |
| 35. |  | there are few grammatical errors such as " what facilitates or hinder" | **We have checked for grammatical errors** | 1-27 |
